# Understanding the drivers of modern contraceptive use trend change and inequalities among sexually active women in Lesotho: A decomposition analysis (2004–2023)

**DOI:** 10.1371/journal.pone.0352594

**Published:** 2026-06-26

**Authors:** Million Phiri, Mapitso Lebuso, Nthatisi Leseba, Talent Tapera, Joseph Kazibwe, Nebechukwu Henry Ugwu, Garikayi Bernard Chemhaka

**Affiliations:** 1 Department of Demography, Population Sciences and Monitoring and Evaluation, School of Humanities and Social Sciences, University of Zambia, Lusaka, Zambia; 2 Demography and Population Studies Programme, Schools of Public Health and Social Sciences, University of the Witwatersrand, Johannesburg, South Africa; 3 Centre for Social Development in Africa, Schools of Social Sciences, University of Johannesburg, Johannesburg, South Africa; 4 Department of Statistics and Demography, Faculty of Social Sciences, National University of Lesotho, Maseru, Lesotho; 5 College of Graduate Studies, University of South Africa, Pretoria, South Africa; 6 Department of Clinical Science, Malmö, Lund University, Malmö, Sweden; 7 Department of Sociology and Anthropology, Faculty of Social Sciences, University of Nigeria, Nsukka, Enugu, Nigeria; 8 Rehabilitation and Health Services Department, College of Health and Public Service, University of North Texas, Denton, Texas, United States of America; University of Salamanca, SPAIN

## Abstract

**Background:**

Improving utilization of contraceptive methods serves as a powerful catalyst for advancing women’s education, health, and personal autonomy. Many countries have pledged to enhance contraceptive availability as part of their commitment to the Sustainable Development Goals (SDGs), specifically under indicator 3.7.1. This study sought to understand the drivers of modern contraceptive use trend and inequalities among sexually active women of reproductive ages in Lesotho during the period 2004–2023.

**Methods:**

The study used four rounds of Lesotho demographic and health survey datasets for the years 2004, 2009, 2014 and 2023. A total sample of 22,325 sexually active women of reproductive ages 15–49 years was used in the analysis. Blinder-Oaxaca decomposition technique was used to analyse drivers of trend change in modern contraceptive while the concentration curves and indices were used to assess the levels of inequality. All analyses were conducted in Stata software version 17 and weighted to account for complex sampling techniques in demographic and health surveys.

**Results:**

The prevalence of modern contraceptive use among sexually active women increased significantly from 36.1% (95% CI: 34.2, 37.9) to 62.7% (95% CI: 61.1, 64.2) during the period 2004–2023. Overall, about 10% of the trend increase in modern contraceptive use was attributable to changes in the compositional characteristics of women. On the other hand, 90% of the trend increase was due to contraceptive behaviour change among sexually active women. Changes in the proportion of women with tertiary education (22.41%) emerged as the major contributor to trend increase in modern contraceptive use. The concentration curves showed that utilization of modern contraceptive methods was profoundly higher among the wealthy than the poor in the first three rounds of the surveys and disappeared in the last round (2023). This pro-rich inequality was persisted throughout the study period except for 2023 where it disappeared. Erreygers concentration Index (EI) values were 0.2360 in 2004, 0.1606 in 2009, 0.0473 in 2014 and −0.0164 in 2023.

**Conclusion:**

The observed reduction in inequality in modern contraceptive use in Lesotho from 2004 to 2023 reflects meaningful progress in women’s reproductive autonomy. This could be explained by improvements in education, health service access, empowerment, and social change. Sustaining these gains will depend on continued investments in the health system, educational opportunities, and community-engaged approaches that are culturally sensitive to respond to the contraceptive barriers and shifting demographic realities.

## Introduction

Contraception is widely recognised as a key component of reproductive health, offering significant benefits for maternal and child well-being, gender equality, and socioeconomic development [1–3]. Over recent decades, various international conferences and policy frameworks have been implemented to address family planning challenges and promote universal access to contraceptive services. These include the 1994 International Conference on Population and Development (ICPD), the Sustainable Development Goals (Goal 3.7.1), and the WHO Global Strategy for Sexual and Reproductive Health, and the Maputo Plan of Action (2016–2030) reinforce these global commitments by advocating for equitable access to comprehensive sexual and reproductive health services, including modern contraception [4–7]. At the national level, many countries-including Lesotho, through its National Strategic Development Plan II (2018/19–2022/23) and Reproductive, Maternal, New born, Child, and Adolescent Health (RMNCAH) strategy have prioritised family planning as a key strategy to reduce unmet need and improve health outcomes [8,9].

Globally, contraceptive use has increased but remains low in Sub-Saharan Africa [3,10,11]. Among the 1.9 billion sexually active women of reproductive age (15–49 years) in 2021, 1.1 billion needed family planning; of these, 874 million were using contraceptive methods, and 614 million had an unmet need [4,12]. These figures mark an improvement from 2019, when 842 million sexually active women used contraceptive methods and 270 million had an unmet need [4]. Despite this progress, Sub-Saharan Africa continues to have lower contraceptive prevalence rates, reflecting regional disparities in access and utilization [13,14].

In Lesotho, contraceptive use among married women of reproductive age has shown consistent improvement over the past two decades, rising from 35% in 2004 to 65% in 2023 [15]. Despite these gains, significant disparities persist in the country. [15]. Studies done in other countries in SSA also highlight that inequality in contraceptive access and use remains one of the main obstacles to achieving universal reproductive health coverage [16–19]. Evidence from both national surveys and regional studies across Sub-Saharan Africa indicates that uptake is uneven across socioeconomic and demographic groups. Poorer, less-educated, rural, and younger women consistently exhibit lower rates of contraceptive use than their wealthier, urban, and more educated counterparts [16,18,19].

Education plays a particularly critical role in shaping contraceptive behaviour, as it enhances women’s knowledge and awareness of available methods, dispels misconceptions, and promotes autonomy in reproductive decision-making [18]. Furthermore, educated women are more likely to participate in the workforce, where effective fertility control and child spacing are essential to maintain career stability and avoid employment disruptions [18]. Cultural and religious norms also play a substantial role in influencing contraceptive behaviour. In some settings, patriarchal expectations, religious prohibitions, and the requirement for spousal consent to access family planning services continue to undermine women’s autonomy and impede contraceptive uptake [20–22].

While the benefits of modern contraception are well established, equitable access to family planning services remains a persistent challenge in Lesotho. These disparities underscore the need for a deeper understanding of the structural and contextual factors shaping contraceptive use. Therefore, this study conducted to answer the research question: how have trends changed and what socioeconomic inequalities exist in modern contraceptive use among sexually active women of reproductive age in Lesotho? To achieve this, the following were the research objectives; (i) to examine the trends in modern contraceptive use among sexually active women of reproductive age in Lesotho and (ii) To assess socioeconomic inequalities in modern contraceptive use among sexually active women of reproductive age in Lesotho. The application of decomposition analyses techniques makes it possible to identify the key drivers of observed changes over time [23–25]. Consequently, this approach provides a robust framework for providing family planning information to guide programme and policy decision making at the country and regional levels.

### Theoretical framework

The study’s theoretical framework draws on the Andersen Behavioral Model of Health Services Use to examine the factors influencing transitions in modern contraceptive use among sexually active women in Lesotho. The Anderson model explains that an individual’s utilization of health services is a function of factors that predispose the use of health service (predisposing), factors that enable the use of health service (enabling) and factors that show the need for the use of health service (need) [26]. Here, modern contraceptive use among women is explained based on factors classified into individual or contextual level, enabling and need factors. The individual factors are those which are peculiar to individuals. For example, wealth index of a household serves as an enabling factor for modern contraceptive use among women because higher economic status improves the ability to afford services, access health facilities, and overcome cost-related barriers to contraception [27–29]. Furthermore, access to information is an enabling factor for modern contraceptive use because exposure to accurate family planning information increases awareness, corrects misconceptions, and facilitates informed decision-making about contraceptive options [30–32].

**Fig 1:**
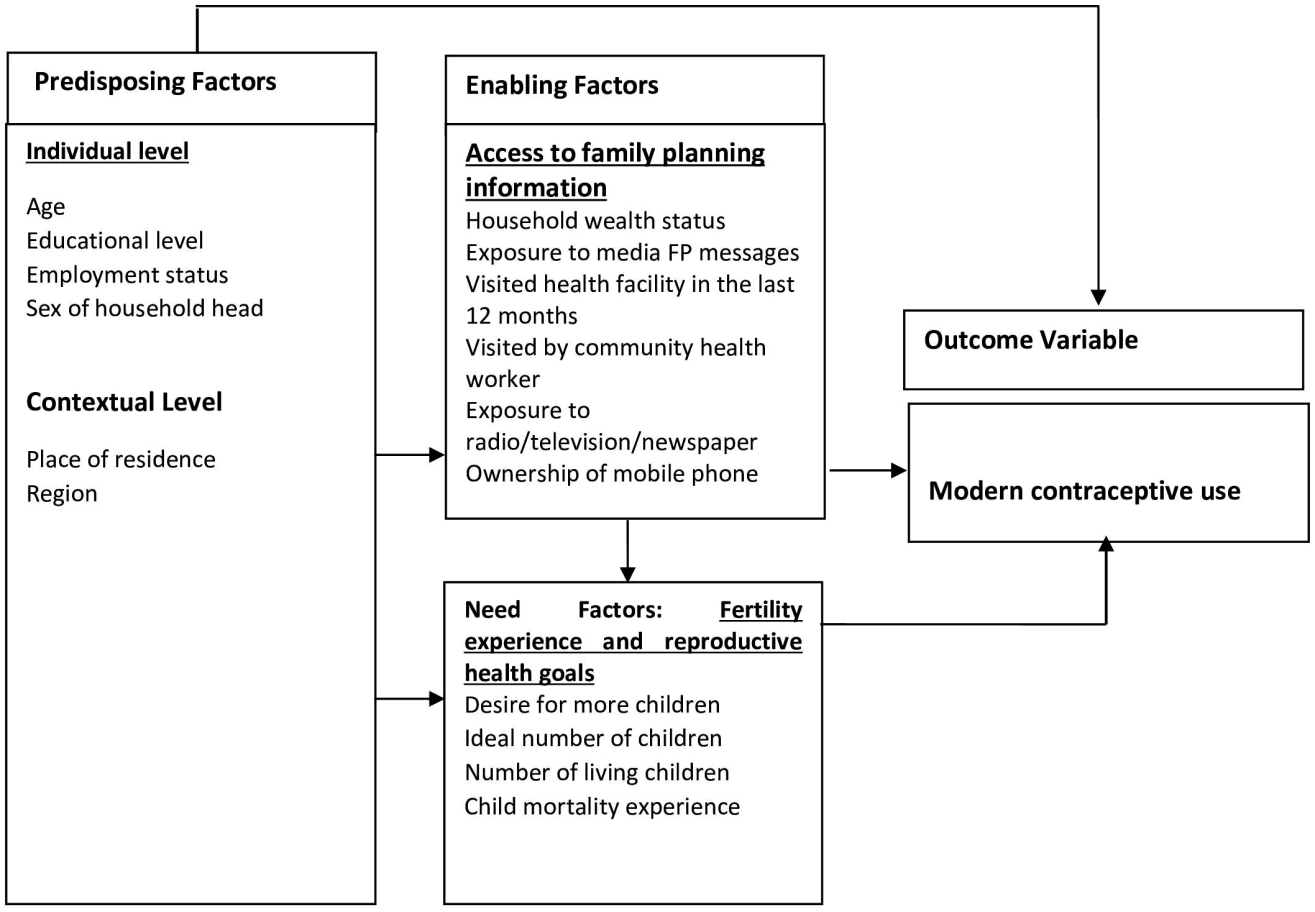
Conceptual Framework showing the determinants of contraceptive use at both individual and community-level adapted and modified from Andersen’s Behavioural Model of Health Service Utilization.

On the other hand, parity is considered as need factor because women who have already had children are more likely to desire spacing or limiting births, thereby increasing their demand for contraception [32,33]. Contextual factors refer to conditions existing outside the individual that can shape health behaviour, such as place of residence and region. Although similar in nature to individual characteristics, these factors are defined and measured at the community level [26]. Contextual factors can shape individual characteristics, which in turn affect health outcomes, or they may exert a direct influence on health outcomes [34]. Both individual and contextual factors influence contraceptive use through intermediate mechanisms, namely enabling and need factors. In this way, the Andersen Behavioral Model of Health Services Use highlights the interconnections between individual, community-level, and intermediate determinants in shaping women’s use of contraceptive methods, illustrating how these factors collectively inform the decision to adopt modern contraception. (Fig 1). Prior studies have shown how several of these factors at different levels play significant roles in determining the use of contraceptives among women of reproductive age in sub-Saharan Africa and elsewhere [34–38].

## Methods and data

### Data source

**Fig 2:**
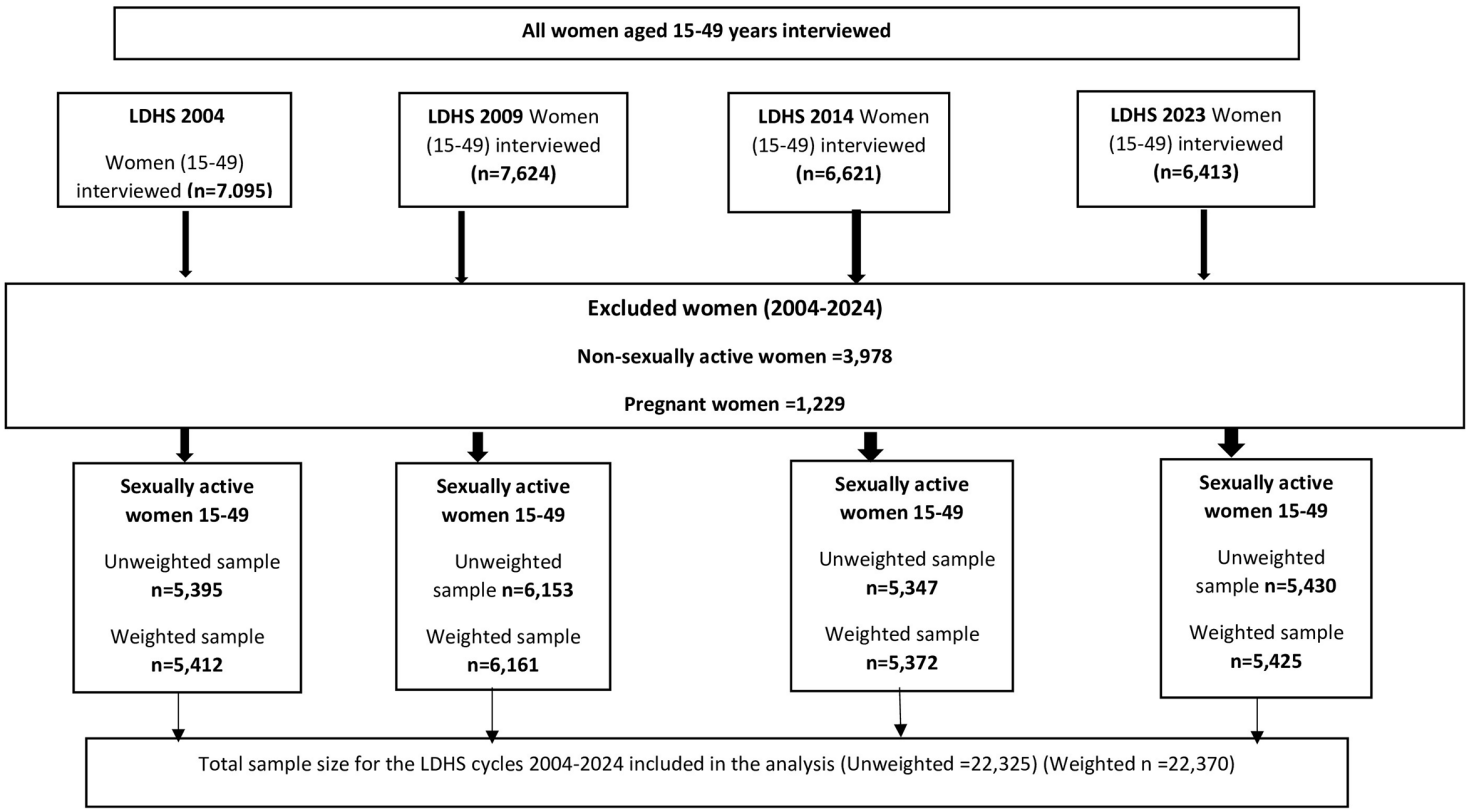
Sample derivation of sexually active women from the 2004-2023 Lesotho Demographic and Health Surveys.

This analysis drew on data from four Lesotho Demographic and Health Surveys (LDHS) conducted between 2004 and 2023, focusing on the women's datasets (IR) which included reproductive-aged women (15–49 years). The LDHS employed a nationally representative, two-stage stratified cluster sampling design, dividing regions into rural and urban areas. The first stage involved selecting enumeration areas (EAs) proportional to the region's size, followed by household listing in the selected clusters. Approximately 25–30 households were selected per cluster [15]. The study included 22,325 sexually active women of reproductive aged 15–49 years who were living in Lesotho at the time of the surveys. We extracted relevant variables from the women's dataset (IR file), focusing on fecund, sexually active women aged 15–49 years from the 2004, 2009, 2014, and 2023 surveys (see Fig 1 for details). The sampling methodology is thoroughly outlined in the LDHS report [15] and other publications [39]. The procedure for the extraction of the analytical sample is outlined in Fig 2.

### Study variables and measurement

#### Dependent variable.

The outcome variable for this study is the use of modern contraceptive methods, measured by the question: “are you currently using any modern method of contraception to prevent pregnancy?” asked to all sexually active women in the DHS. The DHS variable (v364) was used to measure modern contraceptive use. The variable has four response categories (using modern method, using traditional method, intend to use later, does not intend to use). For this analysis, sexually active is defined by the DHS question (v525), “how old were you when you first had sexual intercourse?” Thus, all women who never had sex at the time of the survey classified as non-sexually active. This definition is consistent in all the four LDHS datasets used in this study. Based on the DHS classification, modern contraceptive methods included female sterilization, male sterilization, intrauterine device (IUD), injectables, implants, pills, male and female condoms, and emergency contraception, which are described in detail in previous publications [4,40]. The analysis focused on a subset of women, excluding those who were not sexually active, pregnant, or self-reported as infecund at the time of the survey. A binary outcome variable was created, categorizing sexually active women as either users of modern contraceptive methods coded as “1” or else classified as non-users and coded as “0”.

#### Independent variables.

The independent variables classified in the study were selected based on their relevance in the previous studies as predisposing and enabling factors influencing modern contraceptive usage among women in SSA [10,41–44]. For example research in sub-Saharan Africa has shown that place of residence and region are significantly associated with modern contraceptive use [45].

This study categorizes determinants of modern contraceptive use into three groups. Predisposing factors, which include socio-demographic and cultural characteristics such as age of a woman (15–24 years, 25–34 years, 35–49 years), region (Leribe, Berea, Maseru, Mafeteng, Mohale's Hoek, Quthing, Qasha's Nek, Mokhotlong, Thaba-Tseka), place of residence (urban/rural), and education (no education, primary, secondary, higher). Enabling factors, including wealth index (poor, middle, Rich), Working status (Not working, working), visited health facility in the last 12 months (no, yes), exposure to listening to radio (no, yes), exposure watching television (yes, no), exposure to reading newspaper (no, yes), visited by community health worker (no, yes), media exposure to family planning messages (FP messages) (no, yes), and child mortality experience (no, yes) facilitate contraceptive use. Need factors, such as number of living children (0–1, 2–3, 4–5, 6+), ideal family size (0–1, 2–3, 4–5, 6+), and desire for more children (want another, no more, undecided), reflect the perceived need for contraceptive use.

### Statistical analysis

This study employed a range of statistical analyses, including descriptive, bivariate, and multivariable decomposition analysis. Descriptive analysis was used to assess trends in modern contraceptive use over time, stratified by key predictor variables, and examined separately for each survey phase. Trend analysis was conducted across four LDHS datasets to assess changes between successive survey phases. Blinder-Oaxaca decomposition analysis was used to examine changes in modern contraceptive use among sexually active women over time. Additionally, concentration curves and corrected concentration index (CCI) decomposition analysis was used to investigate inequality in modern contraceptive use between the earliest DHS (2004) and latest DHS (2023) survey phases.

Using concentration curves and the corrected concentration index (CCI) is justified because they allow for a clear and robust assessment of the extent and direction of socioeconomic inequalities in modern contraceptive use over time. The CCI decomposition further helps to identify and quantify the contribution of specific individual and household-level factors to these observed inequalities, providing insight into which determinants drive disparities. Additionally, the Blinder–Oaxaca decomposition is appropriate for analysing drivers of trend increase over time rural) by separating the trend changes into components explained by differences in characteristics and differences in their effects. Together, these methods provide a comprehensive understanding of both trends and drivers of inequality, making them suitable for policy-relevant analysis.

### Decomposition analysis techniques

The decomposition analysis included three steps. (a) concentration curves and corrected concentration index, (b) CCI decomposition and (c) Blinder-Oaxaca decomposition. The detailed description of each methods is outlined below.

(a) Concentration curves and corrected concentration index

We measured inequality in modern contraceptive use using concentration curves and the concentration index. Concentration curves graphically depict the distribution of health benefits across socioeconomic groups by plotting the cumulative proportion of the outcome against the cumulative proportion of the population ranked by wealth index [46]. The wealth index, estimated by DHS, is a composite measure of household living standards based on ownership of assets like household items, transportation, land, and livestock, constructed using principal component analysis [15]. We used concentration curves to assess socioeconomic inequality in outcome variables over time. The curves plot the cumulative percentage of the health variable against the cumulative percentage of the population ranked by wealth index, from poorest to richest. A 45-degree line (line of equality) serves as a reference point: curves above it indicate pro-poor distribution, while curves below it indicate pro-rich distribution [47]. The concentration index (CI) quantifies the degree of inequality, calculated as twice the area between the concentration curve and the line of equality. The CI ranges from −1–1, indicating whether the rich or poor benefit more, or if benefits are equally distributed [46]. The mathematical derivation of the CI is detailed in works by [48]. Given the binary nature of our outcome variables (0,1), the standard concentration index (CI) estimates may be inaccurate due to mean-dependent bounds, limiting comparisons across populations with different health levels [46,47]. To address this, we used the corrected concentration index (CCI), also known as the Erreygers Index (EI), which accounts for the bounded nature of binary variables. We estimated the concentration curve and index using the concentration index command in Stata 18, adjusting for sample clusters, strata, and probability weights to ensure accurate estimates.

(b) CCI decomposition

In this decomposition, we sought to identify the causes of the existing inequality in the outcome variables and estimate the contribution of the independent variables towards the inequalities in the outcome variable of interest [49]. We opted for the general method for decomposing the causes of socioeconomic inequality in health described by Heckley et al., 2016 to identify the causes of the inequality [50]. Heckley et al., 2016 argue that their method for decomposition of inequalities is more suitable in determining the causal effect of a covariate on the inequality index [50]. Furthermore, the method explicitly accounts for the covariance between health outcomes and socioeconomic rank. This provides a more accurate and unbiased estimation of socioeconomic inequalities, particularly when the relationship between health and socioeconomic status is complex or non-linear. The decomposition method by Wagstaff et al. has some important limitations. It is one dimensional focusing on health and ignoring the rank based on the socioeconomic variable. Therefore, the decomposition method by Wagstaff explains only the variation of the health variable but not the covariance between the health variable and rank based on socioeconomic status [50]. The Wagstaff, van Doorslaer, Watanabe (WDW) does not determine the causes of the inequality. Other challenges of the decomposition method by Wagstaff et al. include the difficulty to interpret the results.

The general method for decomposing the causes of socioeconomic inequality in health shows marginal contributions, making it more useful to policymakers who seek to understand the key drivers of inequality in contraceptive coverage. The regression-based decomposition method using Recentered Influence Function (RIF) is considered more reliable for analyzing socioeconomic inequality in health. This approach involves two steps: (i) computing the RIF for the rank-dependent index, and (ii) regressing the RIF on covariates to estimate marginal effects. We applied the RIF-EI-OLS decomposition method to the Erreygers Concentration Index (EI) for modern contraception, and extended it to other indices (CI, ARCI, SRCI, WI) using RIF-I-OLS. The estimated coefficients from the RIF-I-OLS decomposition represent the association between each covariate and its influence on the index, allowing for interpretable results

(c) Blinder-Oaxaca decomposition

We used two component Blinder-Oaxaca multivariable decomposition analysis to identify the determinants of change in trend of contraceptive use between 2004 and 2023. Thus, the analysis focused on how contraceptive use prevalence responds to differences in selected women’s characteristics and how these variables shape the differences across the two survey points. Decomposition analysis aimed to identify the potential sources of variations in the prevalence of modern contraceptive use in Lesotho, over the 19 years’ periods. Multivariable decomposition analysis for the non-linear response model used the output from a logistic regression model since it is a dichotomous variable. The difference in the percentage of contraceptive over time is attributable to the compositional change between the two surveys and the difference in the effects of those selected independent variables. That means the change in contraceptive use is divided into the differences in characteristics (endowment component) and the effect of the selected variables (coefficient component). Besides, the Variance inflation factor (VIF) was applied to check multicollinearity among independent variables. Therefore, there was no multicollinearity between independent variables since the VIF value were less than 5.

### Ethical approval and consent to participate

This study employed data from anonymised, publicly accessible datasets managed by the DHS Program. Authorization and waiver was requested from and approved by the DHS program to obtain the data for research purposes. The DHS surveys underwent rigorous ethical review and received approval from the ICF Institutional Review Board in Lesotho. The data analyzed in this study can be found publicly at (https://dhsprogram.com/). Permission to use DHS datasets was granted by the DHS program. Ethical approval for the DHS was obtained from the Institutional Review Boards (IRBs) of ICF and the Lesotho Ministry of Health Research and Ethics Committee. All approvals were obtained prior to the commencement of data collection exercise for the 2004–2023 LDHS.

## Results

### Description of the study samples

The percent distribution of sample characteristics of all respondents captured in all the four LDHSs are presented in Table 1. Results show that in the first and last surveys (2004 and 2023), most of the women captured were in the age group of 35–49 years (36.1% and 40.3%, respectively) while in the second survey (2009) most women were in the age groups 15–24 and 25–34 years (33.6% for both age groups). Furthermore, findings indicate that in the 2014 survey, most of the women were in the age group 25–34 years (35.5%). Across all the surveys, majority of the respondents were from the rural areas (75.8% (2004), 65.6% (2009), 63.3% (2014), and 54.9% (2023), respectively) showing a decline in the percent of women participating in the survey over the years in rural areas.

**Table 1:** Description of background characteristics of sexually active women in Lesotho, DHS 2004- 2023.

Background Characteristics	DHS 2004	DHS 2009	DHS 2014	DHS 2023
	N = 5,412 (N/%)	N = 6,161 (N/%)	N = 5,372 (N/%)	N = 5,425 (N/%)
**Age**				
15–24	1793 (33.1)	2070 (33.6)	1744 (32.5)	1576 (29.1)
25–34	1665 (30.8)	2072 (33.6)	1909 (35.5)	1663 (30.7)
35–49	1954 (36.1)	2020 (32.8)	1719 (32.0)	2186 (40.3)
**Residence**				
Urban	1311 (24.2)	2118 (34.4)	1974 (36.7)	2446 (45.1)
Rural	4101 (75.8)	4043 (65.6)	3398 (63.3)	2979 (54.9)
**Education level**				
None	127 (17.0)	86 (1.4)	58 (1.1)	34 (0.6)
Primary	3251 (60.0)	3020 (49)	2141 (39.9)	1446 (26.7)
Secondary	1955 (21.0)	2656 (43.1)	2642 (49.2)	2944 (54.3)
Higher	80 (2.0)	400 (6.5)	532 (9.9)	1002 (18.5)
**Household wealth status**				
Poorest	750 (13.9)	858 (13.9)	763 (14.2)	775 (14.3)
Poorer	991 (18.3)	956 (15.5)	837 (15.6)	891 (16.4)
Middle	936 (17.3)	1068 (17.3)	997 (18.6)	1058 (19.5)
Richer	1241 (22.92)	1521 (24.7)	1312 (24.4)	1325 (24.4)
Richest	1449 (27.6))	1758 (28.5)	1463 (27.2)	1378 (25.4)
**Employment status**				
Unemployed	3028 (56.0)	3461 (56.2)	3088 (57.5)	3048 (56.2)
Employed	2381 (44.0)	2699 (43.8)	2285 (42.5)	2377 (43.8)
**Number of living children**				
0-1	2346 (43.4)	3027 (49.1)	2595 (48.3)	2799 (51.6)
2-3	1805 (33.4)	2050 (33.3)	2001 (37.2)	2137 (39.4)
4-5	850 (15.7)	794 (12.9)	591 (11.0)	390 (7.2)
6+	411 (7.6)	291 (4.7)	186 (3.5)	99 (1.8)
**Desire for more children**				
Want more	2126 (40.5)	2414 (40.6)	2254 (42.5)	2171 (40.8)
No more	2984 (56.8)	3525 (58.4)	2979 (56.2)	2990 (56.2)
Undecided	141 (2.7)	92 (1.5)	64 (1.2)	161 (3.0)
**Ideal number of children**				
0-1	495 (9.2)	640 (10.4)	639 (11.9)	768 (14.2)
2-3	2783 (51.4)	3512 (57)	3306 (61.5)	3449 (63.6)
4-5	1669 (30.8)	1683 (27.3)	1234 (23.0)	1040 (19.2)
6+	464 (8.6)	327 (5.3)	193 (3.6)	168 (3.1)
**Experienced child mortality**				
No	4405 (81.4)	4825 (78.3)	4278 (79.6)	4140 (76.3)
Yes	1007 (18.6)	1337 (21.7)	1095 (20.4)	1285 (23.7)
**Exposure to listening radio**				
No	3526 (65.2)	4787 (77.7)	4039 (75.2)	4202 (77.5)
Yes	1885 (34.8)	1371 (22.3)	1333 (24.8)	1223 (22.3)
**Exposure to watching television**				
No	4881 (90.2)	5788 (93.9)	4582 (85.3)	4450 (82.0)
Yes	529 (9.8)	373 (6.1)	791 (14.7)	975 (18.0)
**Exposure to reading newspaper**				
**No**	4767 (88.1)	5548 (90.0)	4755 (88.5)	4885 (90.5)
**Yes**	643 (11.9)	613 (10.0)	617 (11.5)	540 (10.0)
**Visited health facility in the last 12 months**				
**No**	3606 (66.6)	3533 (57.4)	1676 (31.2)	1587 (29.2)
**Yes**	1805 (33.4)	2625 (42.6)	3696 (68.8)	3839 (70.8)
**Visited by community health worker**				
**No**	5164 (95.4)	5910 (96.0)	5083 (94.6)	4743 (87.4)
**Yes**	247 (4.6)	247 (4.0)	289 (5.4)	682 (12.6)
Exposure to media FP messages				
No	3441 (63.6)	4412 (71.6)	3510 (65.3)	3631 (66.9)
Yes	1971 (36.4)	1749 (28.4)	1863 (34.7)	1795 (33.1)

In terms of education level, there was a variation between the years in the level of education. In 2004 and 2009, most of the respondents had primary level of education, 60% to 49% respectively while majority of respondents in the 2014 and 2023 surveys had secondary level of education that is 49.2% to 54.3% respectively. There was an increase in the proportion of respondents with tertiary education over the years.

Across all the years, majority of respondents were not in employment 56.0% in 2004, 56.2% in 2009, 57.5% in 2014 and 56.2% in 2023. Furthermore, the trend in exposure to media FP messages showed a fluctuation over the years with a decline from 36.4% in 2004 to 28.4% in 2009. In the 2014 survey, exposure to media FP messages increased to 34.7%, however, a slight decline was further observed in the 2023 survey to 33.1% (Table 1).

### Modern contraceptive use trends in Lesotho

**Fig 3:**
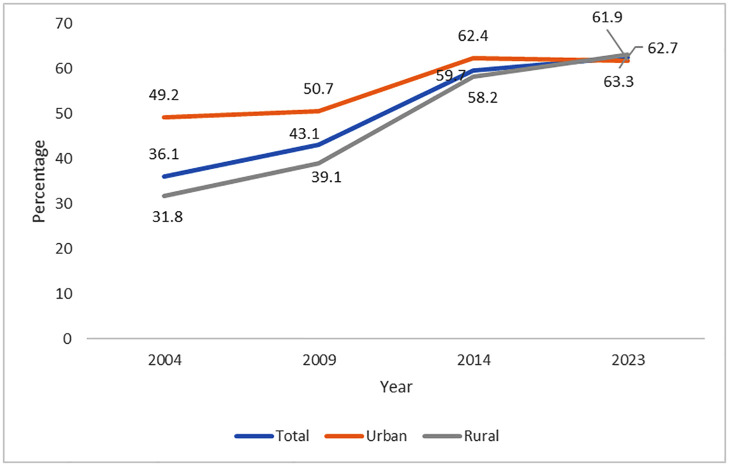
Trends in modern contraceptive use among sexually active women in Lesotho 2004- 2023.

**Fig 4:**
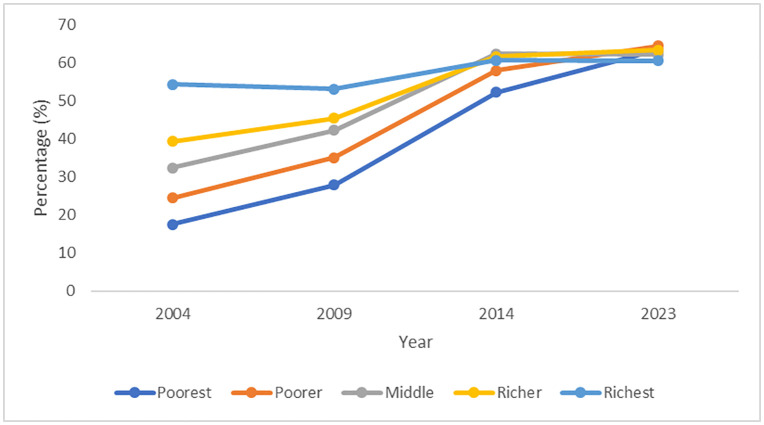
Trend of the proportion of modern contraceptive use by wealth status in Lesotho 2004- 2023.

Overall, the findings show that during the period 2004–2023 modern contraceptive use in Lesotho increased from 36.1% to 62.7%. In urban areas, the prevalence increased from 49.2% in 2004 to 63.3% in 2023. On the other hand, in rural areas, the increase was from 31.8% in 2004 to 61.9% 2023 (Fig 3). Fig 4 shows the proportion of women who using modern contraceptive methods in each wealth quintile over time. There is generally an increasing proportion of women using modern contraception across all wealth strata over time with the poorest and the poorer groups showing the highest increase.

**Fig 5:**
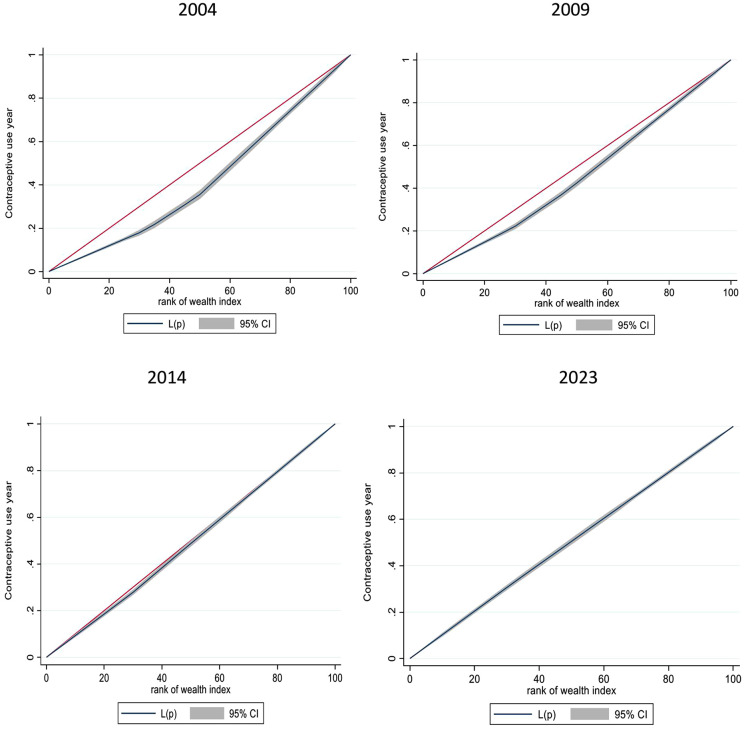
Inequality trends in utilization of modern contraceptives by wealth status (2004 −2023).

Fig 5 shows four concentration curves for the years 2004, 2009, 2014 and 2023 showing the extent of inequality in utilization of modern contraceptive methods among the different wealth groups. The three concentration curves for 2004, 2009 and 2014 have the curve below the line of equality indicating that modern contraceptive utilization was significantly tilted toward respondents from rich households suggesting women in higher wealth ranks used modern contraceptives more than those from lower wealth quintiles. However, the 2023 curve show that wealth quintile inequality in utilization of modern contraceptive use had disappeared, suggesting no significant wealth-related inequality. Overall, the concentration curves show that inequality in utilization of modern contraceptive was more prominent in the years 2004 and 2009.

### Description of contraceptive use transition by socio-demographic characteristics of sexually active women

Trend change in contraceptive use among the respondents varied with background characteristics during the period 2004–2023. The prevalence of contraceptive use increased in most of the categories that were analysed in this study (Table 2). Overall, the prevalence of contraceptive use increased by 26.6% among respondents during the period 2004–2023. Based on age, the most increase in contraceptive use was observed among women aged 35–49 years, 31.0 percentage points in the same period 2004–2023. In terms of residence, women living in rural areas showed the most improvement in percentage change of contraceptive use (31.5% compared to 12.7% in urban areas). Regarding wealth status, variations were observed in terms of contraceptive use change over time in Lesotho. The most increase in contraceptive use by wealth status during the period 2004–2023 was observed among women in the poorest wealth status (45.8%), and the lowest increase was observed among women in the richest wealth status (8.3%).

**Table 2:** Description of modern contraceptive use trends by background characteristics among sexually active women in Lesotho, DHS 2004-2023.

Background Characteristics	DHS 2004 N = 5,412	DHS 2009 N = 6,161	DHS 2014 N = 5,372	DHS 2023 N = 5,425	Percentage point change (2004–2023)
	(N/%)	(N/%)	(N/%)	(N/%)	
**Age**	***	***	***	***	
15–24	566 (31.6)	755 (36.5)	978 (56.1)	906 (57.5)	25.9
25–34	780 (46.8)	1113 (53.7)	1266 (66.3)	1131 (68.0)	21.2
35–49	605 (31.0)	788 (39.0)	963 (56.0)	1362 (62.3)	31.0
**Residence**	***	***	*		
Urban	646 (49.2)	1074 (50.7)	1231 (62.4)	1514 (61.9)	12.7
Rural	1306 (31.8)	1581 (39.1)	1976 (58.1)	1886 (63.3)	31.5
**Education level**	***	***	**	**	
**No education**	10 (8.1)	25 (28.8)	22 (37.3)	9 (27.5)	19.4
Primary	999 (30.7)	1135 (37.6)	1221 (57.0)	895 (61.9)	31.2
Secondary	900 (46.0)	1260 (47.5)	1628 (61.6)	1873 (63.6)	17.6
Higher	42 (53.0)	235 (59.1)	336 (63.2)	623 (62.2)	9.2
**Household wealth status**	***	***	**		
Poorest	132 (17.6)	240 (28.0)	399 (52.3)	491(63.4)	45.8
Poorer	243 (24.5)	336 (35.1)	486 (58.1)	574 (64.5)	40.0
Middle	304 (32.5)	453 (42.4)	624 (62.5)	658 (62.2)	29.7
Richer	489 (39.4)	692 (45.5)	810 (61.7)	840 (63.4)	24.0
Richest	783 (52.4)	935 (53.2)	889 (60.8)	837 (60.7)	8.3
**Employment status**	***	***	***	*	
Unemployed	969 (32.0)	1337 (38.6)	1715 (55.5)	1856 (60.9)	28.9
Employed	982 (41.4)	1318 (48.8)	1492 (65.3)	1543 (64.9)	23.5
**Number of living children**	***	***	***	***	
0-1	769 (32.8)	1141 (37.7)	1395 (53.7)	1522 (54.4)	21.6
2-3	787 (43.6)	1100 (53.7)	1379 (69.0)	1549 (72.5)	28.9
4-5	307 (36.1)	337 (42.5)	357 (60.5)	280 (71.8)	35.7
6+	88 (21.5)	78 (26.8)	76 (40.8)	48 (48.3)	26.8
**Ideal number of children**	***	***	**	*	
0-1	211 (42.5)	251 (39.2)	395 (61.8)	490 (63.8)	21.3
2-3	1104 (39.7)	1610 (45.9)	1991 (60.2)	2180 (63.2)	23.5
4-5	546 (32.7)	700 (41.6)	735 (59.6)	648 (62.4)	29.7
6+	92 (19.7)	94 (28.8)	86 (44.7)	82 (48.4)	28.7
**Desire for more children**	**	**	*	***	
Want more	674 (31.7)	942 (39.2)	1285 (57.0)	1162 (53.5)	21.8
No more	1057 (35.4)	1550 (44.0)	1814 (60.9)	2040 (68.2)	32.8
Undecided	66 (47.3)	32.8 (35.7)	33 (51.1)	93.7 (58.4)	11.1
**Experienced child mortality**	***	***	***	***	
No	1679 (38.1)	2300 (47.7)	2695 (63.0)	2847 (68.8)	30.7
Yes	272 (27.0)	355 (26.6)	512 (46.7)	552 (43.0)	16.0
**Exposure to listening radio**	***	***	**		
No	1150 (32.6)	1964 (41.0)	2357 (58.4)	2595 (61.8)	29.2
Yes	801 (42.5)	692 (50.5)	851 (63.8)	804 (65.7)	23.2
**Exposure to watching television**	***	***	**		
No	1700 (34.8)	2443 (42.2)	2685 (58.6)	2773 (62.3)	27.5
Yes	251 (47.4)	212 (56.9)	522 (66.0)	627 (64.3)	16.9
**Exposure to reading newspaper**	***	**			
No	1642 (34.4)	2337 (42.1)	2822 (59.4)	3056 (62.6)	28.2
Yes	310 (48.1)	318 (51.9)	385 (62.4)	343 (63.5)	15.4
**Visited health facility in the last 12 months**	***	***	***	***	
No	1172 (32.5)	1374 (38.9)	857 (51.1)	885 (55.8)	23.3
Yes	779 (43.2)	1278 (48.7)	2351 (63.6)	2514 (65.6)	22.4
**Visited by community health worker**	*				
No	1841 (35.6)	2540 (43.0)	3018 (59.4)	2992 (63.1)	27.5
Yes	111 (44.9)	111 (45.1)	189 (65.5)	407 (59.7)	14.8
**Exposure to media FP messages**	***	***	**	*	
No	1108 (32.2)	1767 (40.0)	2015 (57.4)	2235 (61.6)	29.4
Yes	843 (42.8)	889 (50.8)	1193 (64.0)	1165 (64.9)	22.1
Total	1951 (36.1)	2655 (43.1)	3207 (59.7)	3399 (62.7)	26.6

*=p<0.05; **=p<0.01; ***p<0.001

In terms of employment status, women without employment recoded the most increase in utilization of contraceptive methods (28.9%) while those in employment recoded the lowest increase in use of contraceptive (23.5%). According to the number of living children, women who had 4–5 children recorded the highest increase in percentage of contraceptive use (35.7%) and the lowest increase was observed among those with 0–1 child (21.6%). Women who reported the ideal number of children as 4–5 children showed the highest increase in utilization of contraceptive methods among women (29.7%) (Table 2).

In terms of experience with child mortality, the most improvement in percentage increase in contraceptive methods use was among women who did not experience child mortality (30.7%). Furthermore, among women who had exposure to media FP messages the percentage increase in use of contraceptive methods was 22.1% between 2004 and 2023 (Table 2).

### Determinants of trend increase in modern contraceptive use Lesotho, 2004–2023

Table 3 presents decomposition analysis results of contraceptive use transition in Lesotho during the period 2004–2023. For endowments or compositional effect, a positive coefficient shows that the increase in the proportion of women possessing that characteristic between 2004 and 2023 was associated with an increase in the utilization of contraceptive methods. A negative coefficient indicates that if the percentage of women with that attribute had remained constant between 2004 and 2023, the prevalence of contraception use in 2023 would have been higher.

**Table 3:** Contribution of explanatory variables to the difference in contraceptive use among sexually active women in Lesotho between 2004-2023, ZDHS.

Background Characteristics	Due to differences in characteristics (E)		Due to differences in coefficients (C)	
	Coefficients	Percent	Coefficients	Percent
**Age**				
15–24	**Ref**		**Ref**	
25–34	0.00015	0.05	−0.02576**	−9.36
35–49	−0.00749***	−2.72	−0.00992	−3.60
**Place of Residence**				
Urban	**Ref**		**Ref**	
Rural	−0.00170	−0.62	0.04482*	16.29
Butha-buthe	**Ref**		**Ref**	
Leribe	−0.00330	−1.20	−0.00918	−3.34
Berea	−0.00254	−0.92	0.00004	0.01
Maseru	−0.00852	−3.09	−0.01437	−5.22
Mafeteng	0.00624	2.27	−0.01729 ***	−6.28
Mohale's hoek	0.00339	1.23	−0.00657	−2.39
Guthing	0.00109	0.40	0.00062	0.22
Qasha's nek	0.00087	0.32	−0.00426 *	−1.55
Mokhotlong	0.00066	0.24	0.00582 *	2.11
Thaba-tseka	−0.00002	−0.01	−0.00135	−0.49
**Woman's education level**				
None	**Ref**		**Ref**	
Primary	−0.09183***	−33.37	0.02312	8.40
Secondary	0.06042***	21.96	0.01151	4.18
Higher	0.06167***	22.41	0.00105	0.38
**Wealth Status**				
Poorest	**Ref**		**Ref**	
Poorer	−0.00011	−0.04	−0.01333	−4.84
Middle	−0.00041	−0.15	−0.02556 ***	−9.29
Richer	−0.00026	−0.09	−0.04825 ***	−17.53
Richest	0.00139	0.50	−0.08820 ***	−32.05
**Working status**				
Unemployed	**Ref**		**Ref**	
Employed	−0.00008	−0.03	−0.00342	−1.24
**Sex of Household Head**				
Male	**Ref**		**Ref**	
Female	−0.00350	−1.27**	−0.01617	−5.88
**Number of living children**				
0-1	**Ref**		**Ref**	
2-3	0.00688***	2.50	0.00573	2.08
4-5	−0.01407***	−5.11	0.00999	3.63
6+	0.00036	0.13	−0.00150	−0.54
**Ideal number of children**				
0-1	**Ref**		**Ref**	
2-3	0.00357	1.30	0.03235	11.76
4-5	0.00204	0.74	0.01631	5.93
6+	0.00896**	3.26	0.00105	0.38
**Desire for more children**				
Want another	**Ref**		**Ref**	
No more	−0.00056	−0.20	0.02832	10.29
Undecided	0.00008	0.03	−0.00183	−0.67
**Exposure to listening radio**				
No	**Ref**		**Ref**	
**Yes**	−0.00141	−0.51	0.02141	7.78
**Exposure to watching television**				
No	**Ref**		**Ref**	
Yes	−0.00246	−0.89	−0.00460	−1.67
**Exposure to reading newspaper**				
No	**Ref**		**Ref**	
Yes	0.00017	0.06	−0.00714	−2.59
**Visited health facility in the last 12 months**				
No	**Ref**		**Ref**	
Yes	0.01807**	6.57	−0.00599	−2.18
**Visited by community health worker**				
No	**Ref**		**Ref**	
Yes	−0.00473**	−1.72	−0.00612**	−2.22
**Exposure to media FP messages**				
No	**Ref**		**Ref**	
Yes	−0.00152	−0.55	0.00903	3.28
**Child mortality experience**				
No	**Ref**		**Ref**	
Yes	−0.00973***	−3.54	−0.01231	−4.47
Constant			0.26142*	95.00
Total	0.02715*	9.87	0.24803***	90.13

*=p<0.05; **=p<0.01; ***p<0.001; Ref=Reference Category

Results show that overall, both changes in compositional structure of women and changes in women's contraceptive behaviour significantly contributed to the increase in the prevalence of contraceptive use in Zambia. The decomposition results showed that the increase in contraceptive use over time was explained by the difference in the selected women’s characteristics and contraceptive behavioural changes between the two survey points. About 10% of the increase in the prevalence of contraceptive use was due to the differences in the composition of women’s characteristics. On the other hand, the change due to the differences in the effect of the selected variables was 90% (Table 3).

Multivariable decomposition analysis of the determinants of transition in contraceptive use has revealed that between 2004 and 2023: maternal education, number of living children, fertility preference, and being visited by community health worker, were the major contributors to trend increase in contraceptive use among sexually active women.

The increment in the proportion of women with secondary level education showed a significant positive contribution of 21.96% to the increase in the contraceptive prevalence rate. Furthermore, an increase in the composition of women who had higher level education contributed about 22.41% to the increase in contraceptive use. The increment in the proportion of women who were visited by a community health worker in the last 12 months and women with no or 1 living child contributed to a significant increase in the prevalence of contraceptive use by 6.57% and 2.50%, respectively. Additionally, a reduction in the proportion of women whose ideal number of children was 6 or more made a significant positive contribution of 3.26% to contraceptive use increase.

### Determinants of inequalities in modern contraceptive use in Lesotho, 2004–2023

Results of inequalities in modern contraceptive utilization among sexually active women in Lesotho as presented in Table 4. Positive values of Erreygers Index signify the presence of inequalities. This indicates that women in the wealthier half of the population significantly use modern contraception more than the less wealthy half, in the years; 2004, 2009 and 2014: concentration index values 0.2360, 0.1605 and 0.0473 respectively. However, the inequalities reduce with time from concentration index 0.2360 in 2004 to −0.0164 in 2023. In 2023, there was no significant wealth-related inequality. Results show that the factors influencing inequalities in modern contraceptive use have changed over time. The inequality in modern contraceptive use in 2004 was significantly influenced by a woman’s age, education level, and household wealth status while in 2023, inequalities were being influenced by district, and sex of household head and number of living children. This shows that there have been changes overtime in terms of factors influencing inequalities in modern contraceptive use in Lesotho.

**Table 4:** Corrected Concentration index (Erreygers Index) for modern contraceptive use among sexually active women in Lesotho (2004-2023).

	2004 (N = 5395)			2009 (N = 6153)			2014 (N = 5347)			2023 (N = 5425)		
	Coefficient	95% confidence interval		Coefficient	95% confidence interval		Coefficient	95% confidence interval		Coefficient	95% confidence interval	
		Lower bound	Upper bound		Lower bound	Upper bound		Lower bound	Upper bound		Lower bound	Upper bound
CCI (EI)	0.2360***			0.1605***			0.0473**			−0.0164		
**Variable**												
**Age**												
15-24	Ref			Ref			Ref			Ref		
25-34	0.1414**	0.0511	0.2317	0.0908	−0.0099	0.1914	−0.0437	−0.1375	0.0501	−0.0014	−0.1138	0.1111
35-49	0.0111	−0.0922	0.1144	0.0009	−0.1121	0.1139	−0.0841	−0.2121	0.0439	−0.0156	−0.1432	0.1119
**Place of residence**												
Urban	Ref			Ref			Ref			Ref		
Rural	−0.0449	−0.1366	0.0468	−0.1000*	−0.1970	−0.0030	−0.0383	−0.1307	0.0541	0.0391	−0.0369	0.1151
**District**												
Butha-buthe	Ref			Ref			Ref			Ref		
Leribe	−0.0192	−0.1585	0.1200	0.0490	−0.1018	0.1998	0.0676	−0.0776	0.2129	0.0623	−0.0514	0.1759
Berea	−0.0192	−0.1585	0.1200	0.0490	−0.1018	0.1998	0.0676	−0.0776	0.2129	0.0623	−0.0514	0.1759
Maseru	−0.0361	−0.1450	0.0727	0.0992	−0.0573	0.2557	−0.0320	−0.1861	0.1221	0.0405	−0.0778	0.1587
Mafeteng	0.0183	−0.1130	0.1496	0.0274	−0.1258	0.1805	0.0663	−0.0926	0.2252	0.1683**	0.0433	0.2933
Mohale's hoek	−0.0398	−0.1412	0.0616	0.1019	−0.0492	0.2530	0.0682	−0.0990	0.2354	−0.0341	−0.1439	0.0757
Guthing	−0.0121	−0.1383	0.1141	0.1499	−0.0185	0.3184	0.1139	−0.0578	0.2856	0.1063	−0.0267	0.2393
Qasha's nek	0.0293	−0.1583	0.2170	0.1347	−0.0174	0.2868	0.0509	−0.1350	0.2368	0.2227**	0.0933	0.3522
Mokhotlong	0.1349	−0.0040	0.2739	0.0801	−0.0928	0.2530	0.0552	−0.1438	0.2543	0.1239	−0.0326	0.2804
Thaba-tseka	0.0709	−0.0552	0.1969	0.0650	−0.1026	0.2327	0.0759	−0.0987	0.2506	0.2069**	0.0765	0.3373
**Education level**												
No education	Ref			Ref			Ref			Ref		
Primary	−0.2571***	−0.3919	−0.1224	−0.0397	−0.3158	0.2363	−0.3593*	−0.6559	−0.0626	−0.2180	−0.6989	0.2629
Secondary	−0.2250**	−0.3903	−0.0596	0.0139	−0.2677	0.2955	−0.3312*	−0.6324	−0.0300	−0.1200	−0.6041	0.3640
Higher	−0.2778	−0.5966	0.0409	0.1388	−0.1867	0.4643	−0.2835	−0.6039	0.0369	−0.0803	−0.5846	0.4239
**Wealth status**												
Poorest	Ref			Ref			Ref			Ref		
Poorer	−0.1605**	−0.2570	−0.0639	−0.1702**	−0.2795	−0.06097	−0.1536*	−0.2814	−0.0056	−0.0353	−0.1414	0.0708
Middle	−0.1911***	−0.2700	−0.1122	−0.1703***	−0.2569	−0.0836	−0.1713**	−0.2765	−0.0654	−0.0252	−0.1129	0.0624
Richer	−0.3127***	−0.4282	−0.1973	−0.27126***	−0.4009	−0.1417	−0.2014**	−0.3191	−0.0609	−0.0130	−0.1417	0.1156
Richest	−0.0708	−0.1943	0.0527	−0.17946*	−0.3162	−0.0427	−0.2603**	−0.4276	−0.1106	−0.1205	−0.2690	0.0281
**Working status**												
Unemployed	Ref			Ref			Ref			Ref		
Employed	0.0068	−0.0580	0.0717	0.0818*	0.0161	0.1474	0.0321	−0.0437	0.1079	0.0576	−0.0315	0.1466
**Sex of household head**												
Male	Ref			Ref			Ref			Ref		
Female	−0.0427	−0.1105	0.0252	−0.0579	−0.1248	0.0089	−0.0556	−0.1315	0.0203	−0.0963*	−0.1702	−0.0224
**Number of living children**												
0-1	Ref			Ref			Ref			Ref		
2-3	−0.0047	−0.0969	0.0875	0.0518	−0.0436	0.1473	0.0003	−0.1010	0.1016	0.0894	−0.0265	0.2053
4-5	0.0004	−0.1174	0.1182	−0.0599	−0.2038	0.0841	0.0306	−0.1246	0.1859	0.0668	−0.1136	0.2471
6+	0.1088	−0.0359	0.2534	0.1087	−0.0582	0.2756	0.1519	−0.0617	0.3655	0.3108*	0.0374	0.5842
**Ideal number of children**												
0-1	Ref			Ref			Ref			Ref		
2-3	−0.0733	−0.1936	0.0470	0.0502	−0.0534	0.1539	−0.0691	−0.1858	0.0476	0.0261	−0.0857	0.1379
4-5	−0.0830	−0.2136	0.0477	−0.0186	−0.1356	0.0985	−0.1194	−0.2564	0.0176	−0.0253	−0.1543	0.1036
6+	−0.0504	−0.1923	0.0915	0.0222	−0.1448	0.1892	−0.0085	−0.2360	0.2190	−0.0729	−0.3141	0.1683
**Desire for more children**												
Want another	Ref			Ref			Ref			Ref		
No more	0.0529	−0.0232	0.1290	0.0320	−0.0427	0.1067	−0.0179	−0.1090	0.0732	0.0984	−0.0106	0.2075
Undecided	0.1960	−0.0588	0.4508	0.1425	−0.0603	0.3454	−0.1956	−0.5155	0.1244	−0.0172	−0.2584	0.2239
**Exposure to listening to Radio**												
No	Ref			Ref			Ref			Ref		
Yes	−0.0328	0.2330	0.2562	−0.0071	−0.1956	0.1814	−0.0350	−0.1617	0.0917	0.0827	−0.1100	0.2754
**Exposure to reading newspaper**												
No	Ref			Ref			Ref			Ref		
Yes	0.0969	−0.0381	0.2318	−0.0038	−0.1599	0.1523	0.0025	−0.1174	0.1225	−0.0458	−0.1939	0.1022
**Exposure to watching Television**												
No	Ref			Ref			Ref			Ref		
Yes	−0.0434	−0.1974	0.1105	0.0694	−0.1061	0.2448	0.0872	−0.0573	0.2316	0.0149	−0.1208	0.1505
**Accessed FP messages via CHW**												
No	Ref			Ref			Ref			Ref		
Yes	0.0064	−0.1421	0.1548	0.0303	−0.1367	0.1972	−0.0834	−0.2202	0.0534	0.0213	−0.1325	0.0899
**Visited health facility in last 12 months**												
No	Ref			Ref			Ref			Ref		
Yes	−0.0134	−0.0850	0.0581	−0.0252	−0.0919	0.0415	0.0201	−0.0500	0.0901	−0.0571	−0.1616	0.0475
**Accessed FP media messages**												
No	Ref			Ref			Ref			Ref		
Yes	0.0186	−0.2550	0.2923	0.0328	−0.1531	0.2187	0.0563	−0.0981	0.2108	−0.0145	−0.1974	0.1684
**Child mortality experience**												
No	Ref			Ref			Ref			Ref		
Yes	−0.0805	−0.1740	0.0131	−0.1636***	−0.2543	−0.0729	−0.1002*	−0.1945	−0.0059	−0.0643	−0.1830	0.0545

* = p < 0.05; ** = p < 0.01; ***p < 0.001; Ref = Reference Category; CCI = Corrected concentration index; EI = Erreygers Index

## Discussion

The study was conducted to examine the drivers of change in trend of modern contraceptive use and assess the trends in inequalities of modern contraceptive use among sexually active women in Lesotho during the period 2004 and 2023. The findings show that behavioural changes among sexually active women was the major contributor to the increase in modern contraceptive in Lesotho. Among compositional factors, the rise in the proportion of women with tertiary education was the largest contributor to the trend increase in modern contraceptive use. Concentration curves revealed persistent pro-rich inequality in contraceptive use across the first three survey rounds, which only disappeared in 2023 when utilization shifted slightly in favour of poorer women. These results are significant in guiding SRH policy and programme direction in the country in order to further increase modern contraceptive utilization.

In line with the study’s theoretical framework, the Andersen Behavioural Model of Health Services Use, we examined how factors operating at multiple levels shape transitions in modern contraceptive use among sexually active women in Lesotho. The model posits that health service utilization is influenced by three broad categories: predisposing factors, which capture individual characteristics such as education and demographic profile; enabling factors, which reflect access to resources and services, including household wealth and exposure to information; and need factors, which represent perceived or evaluated necessity for care, such as fertility preferences, parity, and child survival. Our findings indicate that changes in modern contraceptive use over time were driven predominantly by improvements in predisposing factors, particularly the expansion of secondary and tertiary education among women. This suggests that increased educational attainment may have enhanced knowledge, autonomy, and decision-making capacity, thereby facilitating greater uptake of contraception.

Enabling factors also played a role, although to a lesser extent, likely reflecting gradual improvements in access to services and information. In contrast, need factors—including changes in parity, fertility preferences, and child mortality contributed minimally to the observed changes, indicating that shifts in reproductive intentions alone were insufficient to drive substantial increases in contraceptive use. Overall, these results highlight the interconnected nature of determinants within the Andersen framework and reinforce its applicability in explaining contraceptive use transitions. They further suggest that structural and social changes, particularly those related to women’s education and empowerment, have been more influential than changes in reproductive need in shaping contraceptive behaviour in Lesotho.

Our results suggest that modern contraceptive use among sexually active women in Lesotho has increased remarkably from 36.1% in 2004 to 62.7% in 2023, a trend mirrored across many sub-Saharan African nations over the past two decades [19,24,51–55]. This upward trajectory highlights the cumulative effects of targeted interventions, improved access to reproductive health services, and greater availability of family planning commodities against a backdrop of resistance or slow desire for modern contraceptive uptake [51,56–58]. In Lesotho, our findings between 2004 and 2023 provide evidence that contraceptive uptake is not only increasing overall, but the disparities between wealth status and urban-rural regions are shrinking, indicating successful efforts to reduce geographic and wealth disparities. These trends underscore the impact of sustained national efforts promoting modern contraceptives to address reproductive health challenges, particularly in resource-constrained settings [19,59–62]. Such progress is particularly notable in the context of high HIV prevalence in Lesotho, where integrated services have likely contributed to enhanced contraceptive adoption among the underserved populations [63,64].

At the national level, Lesotho has implemented several policy and programmatic reforms that likely contributed to the observed increases in modern contraceptive use, particularly among rural and poorer populations. The 2017 National Family Planning Guidelines for Service Providers, together with the National Strategic Development Plan II, emphasized equitable and inclusive access to reproductive health services, with a strong focus on underserved communities [8,15,63,64]. In addition, the country’s Reproductive, Maternal, Newborn, Child, and Adolescent Health Strategy supported the expansion of integrated service delivery, including the incorporation of family planning into HIV and maternal health services [8,15,63,64]. These efforts were complemented by the strengthening of community health worker programmes, which improved outreach, health education, and service delivery in hard-to-reach areas. Collectively, these health system and policy initiatives may explain the marked improvements in contraceptive uptake, particularly the rapid catch-up observed among rural and lower socioeconomic groups between 2014 and 2023, reflecting the effectiveness of pro-equity and decentralized approaches to service provision.

The dominance of behavioural change in driving contraceptive uptake, as observed in Lesotho, where it accounted for 90% of the increase, is a common thread in decomposition studies across the region. Contraceptive behaviour change refers to a shift in individuals’ use, non-use, or choice of contraceptive methods over time in response to changes in knowledge, preferences, social norms, or access to services [65]. The coefficients component or Contraceptive behaviour change reflects changes in the effects of observed characteristics as well as the influence of unmeasured factors such as norms, policies, and service quality, rather than purely individual behavioural change. Contraceptive behaviour changes measure in Zambia, Phiri et al. (2024) found that 85% of the CPR increase was attributable to behavioural shifts, largely credited to government policies and improved family planning programs. Similarly, in Rwanda, Kalinda et al. (2022) reported that 76.26% of the rise in mCPR was due to behavioural changes, particularly among sexually active women with primary education and those with 1–2 living children. This pattern is consistent with studies from Tanzania [66] and Ethiopia [55], where behavioural coefficients explained 87.5%, 93.0%, and 66% of the increases, respectively. This consistent finding underscores that, beyond changing population demographics, a fundamental shift in the acceptance and uptake of contraception at the individual level is the primary engine of progress in sub-Saharan Africa.

The observed changes in the effects of the coefficients of contraceptive behaviour change may reflect broader structural and programmatic shifts over time in the country. In particular, SRH policy reforms aimed at expanding access to family planning services. This includes the decentralization of SRH services and the integration of contraception with HIV and maternal health programs, may have strengthened the influence behaviour towards contraceptive use [8,15,63,64]. For example, the integration of family planning into HIV services could have improved access for women already engaged in care, thereby altering utilisation patterns. In addition, shifts in social norms such as increased acceptance of contraception, delayed childbearing, and greater female autonomy may have contributed to changes in how individual and household characteristics translate into contraceptive behaviour. These findings suggest that the observed behavioural component likely captures not only individual-level changes but also the cumulative effects of policy interventions, health system improvements, and evolving sociocultural contexts.

The results suggest that wealth plays an important role in contraceptive use, primarily through changes in the contraceptive behaviour of sexually active women who belong to wealthy household. This reflects the strength of its association with contraceptive uptake rather than shifts in the wealth distribution itself over time. While increases in use are observed among the poor and middle quintiles, these patterns should be interpreted cautiously and do not necessarily indicate a strong pro-poor redistribution. This finding is consistent with studies showing that the effect of wealth on modern contraceptive use can vary across contexts, with improvements among lower quintiles often linked to targeted interventions such as subsidized programs, expanded method choice, and the integration of family planning with maternal healthcare services [60,62,67,68], however this socioeconomic gap cannot be discounted in many African nations [27,62].

The reduction of wealth-based inequality in Lesotho, culminating in the disappearance of the pro-rich bias by 2023, is a significant achievement. This finding aligns with the pro-poor trends observed in Zambia, where Kazibwe et al. (2024) documented a decrease in pro-rich inequality in mCPR from 2007 to 2018. Our results also resonate with the conclusions of Anyatonwu & San Sebastián (2022) in Nigeria, who identified household wealth and women's education as the largest contributors to rural-urban disparities. The successful narrowing of the wealth gap in Lesotho suggests that policies aimed at economic empowerment and reducing structural socioeconomic inequalities, as called for in the Nigerian context, are crucial for equitable contraceptive access.

Our results from decomposition analysis offer some clarity on the drivers of this transition. About 10% of the increase in modern contraceptive use was attributed to compositional changes in women’s characteristics, while 90% to behavioural shifts, emphasizing the role of effect modifications in driving trends as depicted in similar studies [53,69,70]. Education emerged as a major contributor to changes in population composition with increases in secondary and higher education among women, which accounted for over 40% of the gains in contraceptive prevalence in Lesotho. Changes in sexually active women’s composition according to parity, fertility preference, and visits to a health facility were also notable sources of increase in modern contraceptive use. Evidence in low-income countries suggests that education empowers women with knowledge, fosters critical thinking about reproductive choices, and raises awareness about modern methods, all of which are essential for informed decision-making [56,71–73]. The dramatic rise in proportions of women with at least secondary education underscores the importance of sustained investment in girls’ education for public health outcomes, which has been beneficial for most African nations.

The critical role of education as a compositional factor in our study is strongly supported by regional evidence. In Zambia, Phiri et al. (2024) found that an increase in women's secondary education contributed 5.87% to the mCPR rise among sexually active women. Similarly, in Rwanda, Kalinda et al. (2022) reported that improvements in secondary education contributed 7.36% to the increase in mCPR of sexually active women. The study in Cameroon by Pillai & Teboh (2011) also highlighted secondary/higher education as a key compositional driver [74]. Furthermore, the positive contribution of changes in fertility preferences in Lesotho mirrors findings from Zambia, where a reduction in the desire for 6 or more children was a significant factor [53]. This consistency highlights that investments in female education and programs that support smaller family norms create a foundational shift that enables increased contraceptive use.

Nine-tenths of the increase in modern contraceptive uptake was mostly due to changes in uptake among rural sexually active women. A key feature of this increase is the sharp rise in rural areas (from 31.8% to 61.9%) compared to urban settings (from 49.2% to 63.3%), indicating successful efforts to reduce geographic disparities in the study period. This pattern aligns with observations in other sub-Saharan countries, where rural expansions in public health services have led contraceptive gains among underserved populations [55].

The remarkable catch-up by rural sexually active women in Lesotho is a testament to the potential of community-based health strategies. This finding is powerfully echoed in studies from Ethiopia. Mekonnen (2012) attributed 69% of the contraceptive increase among sexually active women in Ethiopia to compositional changes, which were significantly driven by home visits from Health Extension Workers [75]. Similarly, Worku et al. (2015) credited Ethiopia's Health Extension Program as a key driver of behavioural change, particularly among the rural population. The significant positive contribution of being visited by a community health worker in Lesotho's decomposition analysis (6.57%) further reinforces the pivotal role of these frontline health workers in bridging the geographic access gap, a strategy that has proven effective in multiple national contexts.

Despite substantial progress, the decomposition analysis reveals persistent challenges and negative contributions from certain characteristics. The negative coefficients for older sexually active women, primary education, experience of child mortality, and higher parity (4–5 children) indicate that these subgroups remain less likely to adopt modern contraception, pointing to entrenched barriers such as social conservatism and informational deficits [51,56–58]. More intensively targeted interventions may be required to overcome these obstacles and ensure sustained, equitable access.

We found inequalities in modern contraceptive utilization, as quantified by the Erreygers Index, declining from 0.2360 in 2004 to −0.0164 in 2023, suggesting a marked reduction in wealth-based disparities in utilization of modern contraceptives among sexually active women. Concentration curves below the equality line in earlier years from 2004 indicate pro-rich biases [76], which disappeared by 2023, consistent with recent trends in reduced socio-economic status gaps in contraceptive use for some sub-Saharan African countries [77]. This evolution reflects broader socioeconomic improvements contributing to equitable family planning access for most nations in the region. However, the persistence of some residual inequalities underscores the complexity of achieving complete parity across wealth strata [27,78]. These results highlight the success of policies aimed at mitigating economic barriers to reproductive health services in Lesotho.

The evolving nature of inequalities, with determinants shifting from wealth and education to geographic and household factors, underscores the need for dynamic policy responses. While Lesotho has made great strides in reducing wealth-based inequality, the persistent disparities linked to district and female-headed households in 2023 highlight a new frontier for equity programming. This resonates with the complex picture in Nigeria, where Babalola & Oyenubi (2018) explained North-South differentials through a combination of socioeconomic (42.6%) and ideational (42.0%) factors, alongside conjugal dynamics and Islamic culture [79]. This suggests that as structural economic barriers are reduced, deeper cultural, regional, and household-level barriers may become more apparent, requiring multi-pronged approaches that work with religious leaders and address gender dynamics, as recommended in the Nigerian context [79].

The pattern of factors associated with inequality appears to have shifted over time, from age, education, and wealth in 2004 to age, district, and sex of the household head in 2023, suggesting changing associations with contraceptive use patterns. However, these findings should be interpreted cautiously, as the RIF regression coefficients reflect associations with the inequality index rather than causal effects. In earlier periods, socioeconomic factors dominated, aligning with findings from sub-Saharan Africa, where wealth and education significantly affected contraceptive behaviours [4]. By 2023, geographic and household dynamics emerged, reflecting decentralized health systems and women's empowerment in Lesotho. This transition parallels observations in Ethiopian communities, where contextual factors increasingly shape access [80]. Addressing these evolving elements will be essential for sustaining equity gains in family planning.

### Policy implication

The findings point to the need for more targeted and context-specific policy responses in Lesotho to sustain recent gains in modern contraceptive use while addressing emerging inequalities. Although wealth-based disparities have largely been eliminated, residual inequalities linked to district of residence, household characteristics, and higher parity have emerged. This suggests that the next phase of interventions should prioritize geographically targeted strategies focusing on underperforming regions and vulnerable subgroups and tailored outreach to older and high-parity women. Furthermore, strengthening community health worker programmes remains critical for maintaining equitable access, particularly in underserved and rural districts. This should goes hand in hand with continued investment in girls’ education and health system infrastructure. In addition, SRH policies should incorporate culturally sensitive, community-engaged approaches including the involvement of men and local leaders to address persistent sociocultural barriers. Such a balanced approach, combining broad structural investments with targeted interventions, will be essential for advancing universal and equitable access to family planning.

### Strengths and limitations

The present study’s strengths stem from its use of four nationally representative LDHS data over two decades, facilitating robust trend and decomposition analyses comparable to regional studies. The surveys’ high response rates, standardized methodologies, and national coverage enhance reliability. The study’s focus on sexually active women improves relevance to the unmet need for family planning.

However, several limitations warrant careful consideration. First, the cross-sectional nature of the DHS data limits the ability to establish causal relationships, and the observed associations may be influenced by residual confounding, omitted variables, or potential reverse causality. Second, key constructs such as access to services, quality of care, and comprehensive knowledge of family planning are not directly measured in the survey, which may result in incomplete capture of important determinants. Third, reliance on self-reported contraceptive use introduces the possibility of reporting bias, including under- or overestimation due to recall errors or social desirability, particularly among unmarried or adolescent women. In addition, the use of secondary data constrains variable selection and limits the inclusion of context-specific factors.

Methodologically, decomposition techniques are sensitive to model specification, choice of reference groups, and accurate coefficient estimation, and may not fully capture non-linear relationships or unobserved heterogeneity in contraceptive decision-making. Furthermore, differences in survey timing and contextual changes across survey rounds may affect comparability over time. Despite these limitations, the findings provide useful insights into the relative importance of compositional and behavioural factors in shaping contraceptive use patterns in Lesotho, underscoring the need for multifaceted interventions.

## Conclusion and recommendations

In conclusion, the observed rise in modern contraceptive use in Lesotho from 2004 to 2023 reflects meaningful progress in women’s reproductive autonomy necessitated by improvements in education, health service access, empowerment, and social change. Sustaining these gains will depend on continued investments in health system infrastructure, educational opportunities, and culturally sensitive, community-engaged approaches that respond to nuanced barriers and shifting demographic realities. A landmark achievement for Lesotho is the elimination of wealth-based inequality in contraceptive use by 2023, a goal that many neighbouring countries are still striving towards. This equity gains underscores the effectiveness of pro-poor health policies and the decentralization of services. However, the battle for universal access is not over. Persistent negative contributions from factors such as higher parity and the emergence of geographic (district) and household-level determinants of inequality highlight the need for the next phase of interventions to be more nuanced and targeted. To sustain and build upon these gains, future strategies must be multifaceted. Continued investment in girls’ education remains paramount. Health systems must be strengthened to maintain the reach and quality of community health worker programs, which have proven indispensable for rural populations. Policy must also evolve to address the newly identified barriers, targeting specific districts and supporting women in female-headed households through tailored programs. Furthermore, engaging men and community leaders to shift deep-seated cultural and ideational barriers, as suggested by research in other SSA countries, will be crucial. By combining sustained investment in structural enablers with targeted, culturally sensitive programming, Lesotho can secure its gains and continue its trajectory towards universal and equitable access to family planning for all sexually active women.
