# Supplementary material for: Understanding the drivers of modern contraceptive use trend change and inequalities among sexually active women in Lesotho: A decomposition analysis (2004–2023)
Source: PLoS One. 2026 Jun 26;21(6):e0352594. doi: 10.1371/journal.pone.0352594 (PMC13308849; doi:10.1371/journal.pone.0352594)
Supplement: Attachment — Submitted filename: PLOS ONE REVIEWS.docx [file pone.0352594.s001.docx]

PLOS ONE REVIEWS

Under data source, I recommend removal of how data was collected because authors used the secondary data.

Under the independent variables, paragraph 2, the following corrections of place must be considered:

Mohale’s hoek = Mohale’s **H**oek

Guthing = **Q**uthing

Qasha’s nek = Qa**ch**a’s **N**ek

Thaba-tseka = Thaba-**T**seka

The subheading, conclusion, can merged with recommendations in line with the content of the paragraph. Therefore, it will be “ Conclusion and Recommendations”
