# Supplementary material for: Understanding the drivers of modern contraceptive use trend change and inequalities among sexually active women in Lesotho: A decomposition analysis (2004–2023)
Source: PLoS One. 2026 Jun 26;21(6):e0352594. doi: 10.1371/journal.pone.0352594 (PMC13308849; doi:10.1371/journal.pone.0352594)
Supplement: Attachment — Submitted filename: Response to Reviewers.docx [file pone.0352594.s002.docx]

The Editor,

PLOS ONE

20^th^ April 2026

**Sub: Response to Decision Letter: Manuscript ID:** PONE-D-25-62366**:** Understanding the Drivers of Modern Contraceptive Use Trend Change and Inequalities Among Sexually Active Women in Lesotho: A Decomposition Analysis (2004–2023)

Refer to the above subject matter.

We are grateful to the editor and reviewers for their time and constructive comments on our manuscript. We have taken into consideration the second round of rviews and suggestions the editor, and we would like to submit a revised version of the manuscript for consideration by the journal. To reflect changes or amendments made in the revised version of the manuscript, changes or amendments have been highlighted in the initial version of the manuscript. In addition, we provide a point-by-point response explaining how we have addressed the editors comments in the section following this one. We are looking forward to hearing the results of your evaluation.

Yours sincerely,

Lead author

Million Phiri (PhD)

On behalf of co-authors

**Title:** Understanding the Drivers of Modern Contraceptive Use Trend Change and Inequalities Among Sexually Active Women in Lesotho: A Decomposition Analysis (2004–2023)

| **Journal Requirements** | | |
| --- | --- | --- |
| **Editors Remarks** | **Authors Response** | **Reference** |
| Thank you for submitting your manuscript to PLOS ONE. After careful consideration, we feel that it has merit but does not fully meet PLOS ONE’s publication criteria as it currently stands. Therefore, we invite you to submit a revised version of the manuscript that addresses the points raised during the review process. | We would like to extend our gratitude to the editor and reviewers for their valuable time and constructive feedback provided on our manuscript. We have diligently worked towards addressing all the raised comments and have incorporated the suggested edits accordingly. We eagerly anticipate any additional support or guidance moving forward. |  |
| Journal Requirements:  When submitting your revision, we need you to address these additional requirements.  1. Please ensure that your manuscript meets PLOS ONE's style requirements, including those for file naming. The PLOS ONE style templates can be found at  <https://journals.plos.org/plosone/s/file?id=wjVg/PLOSOne_formatting_sample_main_body.pdf> and  <https://journals.plos.org/plosone/s/file?id=ba62/PLOSOne_formatting_sample_title_authors_affiliations.pdf> | We have ensured that all appropriate journal guideline have been followed in presenting the manuscript. | Whole manuscript document |
| 2. Please include your full ethics statement in the ‘Methods’ section of your manuscript file. In your statement, please include the full name of the IRB or ethics committee who approved or waived your study, as well as whether or not you obtained informed written or verbal consent. If consent was waived for your study, please include this information in your statement as well. | The ethics statement has been included in the methods section | Line 411 to 416 |
| 3. Please upload a copy of Figure 1, to which you refer in your text on page 5, 6, 11, 12. If the figure is no longer to be included as part of the submission please remove all reference to it within the text. | The corrections have been made Figure 1 is on page 6 (Conceptial framework). The figure 1 in the results section has been chnaged to Figure 3. All the figures are now correctly numbered | Figures |
| 4. If the reviewer comments include a recommendation to cite specific previously published works, please review and evaluate these publications to determine whether they are relevant and should be cited. There is no requirement to cite these works unless the editor has indicated otherwise. | Thenk you for the guidance. No request has been made to cite specific published works |  |
| **Reviewer 1** | | |
| **Questions and Comments** | **Authors Response** | **Reference** |
| 1) The title and abstract emphasize "sexually active women," which is a crucial and relevant sample. However, the Introduction and parts of the Discussion frequently cite national mCPR statistics for "all women" or "married women" from the LDHS reports. This creates a slight disconnect. The manuscript would be strengthened by consistently framing the entire narrative around the specific population of interest (sexually active women) and clarifying how/why trends for this group might differ from national averages | Thank you for the guidance. All statistics cited reflect those of sexually active women. Furthermore, all studies used in the literature review were those whose analysis group was sexually active women. | Introduction and discussion sections |
| 2)The paper’s aims are broadly stated but could be sharpened. At present, the Introduction mixes global FP trends, Lesotho programme context, and general SSA literature without clearly articulating the specific research questions. In the last paragraph of the Introduction, explicitly list 2–3 concrete objectives | A research question and two specific objectives have been included in the last paragraph of the introduction. | **Line 137-141** |
| 3)You classify covariates into “predisposing”, “enabling”, and “need” factors but do not explicitly cite or connect this to a conceptual framework (e.g., Andersen’s behavioral model). This weakens the health services / health economics framing. Explicitly state that you use Andersen’s model (or similar) to structure covariates, and briefly justify the classification (e.g. wealth as enabling, parity as need, etc.), with a reference. | Thank you for the guidance. A theoretical framework has been included and provides detail of variable classification and interrelations of variables to influne the outcome. | **Line 147-222**  **Figure 1** |
| 4)You repeatedly refer to “sexually active women”, but you do not define how sexual activity is operationalised (e.g. “had sex in the past 4 weeks”, “past 3 months”, or “past 12 months”). This is critical because DHS distinguishes between “ever had sex”, “had sex in last 4 weeks”, etc., and your choice affects both prevalence and comparability over time. In Methods, explicitly state the exact DHS variable(s) used (e.g. `v525` or `v536`) and the time window for “sexually active”, and confirm that the definition is comparable across all four surveys.. | Thenk you for the guidance. The operationalization of the concept “sexually active” has been clarified. The content has been added to the outcome variable description. We also conford that the same definition was applied in the four LDHS datasets used in the analysis. | **Line 271-288** |
| 5)The Introduction says: “contraceptive use among sexually active women… rose from 46.2% in 2004… to 72.1% in 2014… 66.5% in 2023/24 [15]”. But your results (Figure 1, Table 2) show 36.1% in 2004 and 62.7% in 2023. These cannot both be correct for the same population and outcome definition | Thank you for the observation.  The statistics have been corrected in the introduction section. In the DHS report, the trend are only presented for the married women (35% to 65%) who are part of the broader group of sexually active women. In our indepth analysis, we focus on sexually active group (36.1% to 62.7%) which groups non married women. The differences are minor and are as result of differen demnominators. | **Line 116-117** |
| 6)The justification for using the Heckley et al. (2016) method over the more common Wagstaff decomposition is appreciated. However, the explanation is dense and could be more accessible. A clearer, concise summary of the key advantage ("it accounts for the covariance between health and socioeconomic rank") in the main text would be helpful. | Thank you. The suggested text to strethen the justification has been added. | **Line 370-374** |
| 7)You list methods included in “modern contraception”, which is good. However, DHS definitions evolve over time (e.g. addition of emergency contraception; classification of Lactational Amenorrhea Method). You should state whether your classification is harmonized across survey waves and, if any recoding was done, how. Add a sentence clarifying whether the list of modern methods is identical in all four LDHS rounds and, if not, how discrepancies were addressed. | The detail has been added in the outcome variable definition. The DHS has a variable v364 which was already constracted by the DHS program to measure modern contraceptive use. The variable is available in all the DHS dataset and it ensure consistency as result of improvement in the defintion over time. | **Line 281-285** |
| 8)In Table 3, the compositional (E) contributions of wealth categories are extremely small (on the order of ±0.0001–0.0014; less than 1% each), yet the Discussion states that “improvements in household wealth contributed significantly” to contraceptive use trends. In contrast, the major compositional contributors are education (especially secondary and higher) and visits to health facilities. Tone down claims about wealth composition driving the trend. Make clear that wealth appears more important in the coefficients component (C)—i.e. changes in the strength of association between wealth and contraceptive use—rather than in shifts in the wealth distribution. | Thank you for the obssrvation. The appropriate edits have been made in the paragraph in the discussion section. | **Line 659-688** |
| 9)The categorization of "Predisposing, Enabling, Need" factors is standard but not fully utilized in the interpretation. The decomposition results could be discussed through this lens to provide a more structured understanding of what types of factors (e.g., enabling vs. predisposing) drove changes. | Thank you for the guidance. A write has been included in the discussin section to disucss the results in line with the lens of the thoeretical model. | **Line 574-585** |
| 10)The Blinder-Oaxaca result that 90% of the increase is due to the "coefficients" component (labeled as "contraceptive behaviour change") is a central finding. This term requires more precise definition and interpretation. Does it reflect changes in the *effect* of characteristics (e.g., the impact of being rural changed over time), changes in unobserved factors (e.g., norms, program quality), or both? The discussion of this point (page 23) is good but should be expanded and linked more explicitly to potential drivers like policy changes, service integration (e.g., with HIV programs), and shifts in social norms. | 1. The term contraceptive hahavior change has been defined in the discussion section 2. The coefficients component or Contraceptive behaviour change reflects changes in the effects of boths observed characteristics and the influence of unmeasured factors. This has been added to the discussion section. 3. A write up has been added to expand and link the contraceptive behaviour change to potential drivers like policy change, social norms and programme improvement. | **Line 626-633**  **Line 645-657** |
| 11)You report EI values of 0.2360, 0.1606, 0.0473, and –0.0164 for 2004, 2009, 2014, and 2023 respectively. You interpret 2023 as “pro poor” and as inequality having “disappeared”. Yet the magnitude is very small and no standard errors/95% CIs are given for the EI itself. Provide standard errors and 95% CI (or p values) for each EI estimate. | 1. The edit has been made on the interpretation for EI for 2023. The interpretation have been updated to reflect no signficane and not pro-poor. 2. The output results for EIs in Stata produces the stanadard errors and p-values. The p-values for the EIs are indicated in Table 4 for each DHS year. The signifcance was only observed in 2004, 2009 and 2024. | **Line 554-555**  **Table 4** |
| 12) Figure 3: The caption mentions "three concentration curves" but four are described (2004, 2009, 2014, 2023). Please correct. Ensure the figure is of publication quality with a clear legend. | The edit has been made. The figures will formatted using Plos pace application during publication phase. | **Line 462** |
| 13) If the 2023 EI is not statistically different from zero, it is more precise to describe it as “no significant wealth-related inequality” rather than “pro-poor inequality”. | The edit has been effected. | **Line 554** |
| 14)The Discussion notes that in 2004 inequality was “significantly influenced” by age, education, and wealth, whereas in 2023 it was “influenced by district, sex of household head and number of living children”. This is consistent with Table 4 but should be interpreted carefully: RIF regression coefficients indicate association with EI, not necessarily causal contribution. Make clearer that these are associations with the index, and avoid strong causal language (“determine”, “cause”). | Thank for the guidance. The edits have made to relect association and not causality | **Line 756-765** |
| 15) There are several internal inconsistencies in descriptive tables that need careful checking. | Thank you for the observations. All the tables have been formatted and aligned. | **Table 1 and 2** |
| 16)The Discussion is rich in cross country comparisons but could be more concise and more directly focused on implications for Lesotho’s policy-making: targeting districts with residual inequality, tailoring outreach to older, high parity women, strengthening community health worker programmes, etc. | A section dedicated to policy implication has been added to the discussion section | **Line 769-780** |
| 17) The discussion appropriately cites regional literature but would benefit from deeper integration of Lesotho's unique context. Brief mentions of HIV integration and community health workers are good starts. Elaborate more on specific national policies (e.g., National Strategic Development Plan II, RMNCAH strategy), programs, or health system changes that might explain the dramatic catch-up in rural areas and among poorer women between 2014 and 2023. | Thank you for the guidance. A paragraph has been included in the discussion section which provides detail of explainations to justfiy the notable improvements in the contraceptive use among rural women between 2014 and 2023. | **Line 611 - 624** |
| 18). The limitations section is adequate but should be expanded. | Thank you the limitations section has been expanded. | **Line 786-809** |
| **Minors** | | |
| • The manuscript is generally understandable but contains numerous grammatical errors, typographical errors, and long, sometimes repetitive paragraphs (especially in the Discussion). | Thank for the guidance. We engaged an English professional editor to edit the whole manuscript. | **Whole manuscript** |
| • Phrases such as “coul dbe explained by” and “las 12 months” should be corrected. | Edits have beeen made | **Line 303** |
| • Several references are problematic. For example, reference [35] is a paper on dissolved organic matter (DOM) in boreal forest soils, which is clearly unrelated to inequality analysis and looks like a miscitation. | The reference has been removed. It was added in relation to analysis medthods used which is “decomposition analysis” | **References** |
| • Boadu (2022) appears more than once in the reference list | The correction have been made. Only one reference now appear in the reference list | **References** |
| • Some in text references (e.g. “WDW”) are not fully defined (presumably Wagstaff, van Doorslaer, Watanabe). | The correction has been made. | **Line 377-378** |
| • Use consistent terms for wealth groups: e.g. “poorest, poorer, middle, richer, richest” throughout, both in text and tables | The corrections have been made. | **All Tables and manuscript document** |
| **Reviewer 2** |  | |
| General comment: The article is original and meets academic writing requirements with clear statistics and description of findings. Only minor (typos) and deletion under the methodology are brought to the attention of authors. | We appreciate your expertise and time to review our manuscript. We have addressed all the comments are guided. We look forward to your further guidance |  |
| Comment1. Under data source, I recommend removal of how data was collected because authors used the secondary data. | Thank you for the guidance. We realised the journal guidelines require that we should provide detailes of how the secondary data was collected in the DHS. Therefore, we can kept the text in line with journal guidelines. | **Methods section** |
| Comment 2: Under the independent variables, paragraph 2, the following corrections of place must be considered:  Mohale’s hoek = Mohale’s **H**oek  Guthing = **Q**uthing  Qasha’s nek = Qa**ch**a’s **N**ek  Thaba-tseka = Thaba-**T**seka | The edits have been made. | **Line 300** |
| Comment 3 The subheading, conclusion, can merged with recommendations in line with the content of the paragraph. Therefore, it will be “ Conclusion and Recommendations” | The edit has been as suggested | **Line 812** |
