# Supplementary material for: Understanding the drivers of modern contraceptive use trend change and inequalities among sexually active women in Lesotho: A decomposition analysis (2004–2023)
Source: PLoS One. 2026 Jun 26;21(6):e0352594. doi: 10.1371/journal.pone.0352594 (PMC13308849; doi:10.1371/journal.pone.0352594)
Supplement: Peer review history

# Peer review: Decision Letter 0

**DOI:** 10.1371/journal.pone.0352594.r001

**Authors:** José Antonio Ortega

22 Feb 2026

PONE-D-25-62366

Understanding the Drivers of Modern Contraceptive Use Trend Change and Inequalities Among Sexually Active Women in Lesotho: A Decomposition Analysis (2004–2023)

PLOS One

Dear Dr. Phiri,

Thank you for submitting your manuscript to PLOS ONE. After careful consideration, we feel that it has merit but does not fully meet PLOS ONE’s publication criteria as it currently stands. Therefore, we invite you to submit a revised version of the manuscript that addresses the points raised during the review process.

Two reviewers have analyzed the manuscript noting lack of detail in the theoretical framework, inconsistencies in reporting and lack of detail on methodological choices in addition to other minor aspects.

Please submit your revised manuscript by Apr 08 2026 11:59PM. If you will need more time than this to complete your revisions, please reply to this message or contact the journal office at plosone@plos.org. When you're ready to submit your revision, log on to https://www.editorialmanager.com/pone/ and select the 'Submissions Needing Revision' folder to locate your manuscript file.

Please include the following items when submitting your revised manuscript:

- A letter that responds to each point raised by the academic editor and reviewer(s). You should upload this letter as a separate file labeled 'Response to Reviewers'.
- A marked-up copy of your manuscript that highlights changes made to the original version. You should upload this as a separate file labeled 'Revised Manuscript with Track Changes'.
- An unmarked version of your revised paper without tracked changes. You should upload this as a separate file labeled 'Manuscript'.

If you would like to make changes to your financial disclosure, please include your updated statement in your cover letter. Guidelines for resubmitting your figure files are available below the reviewer comments at the end of this letter.

If applicable, we recommend that you deposit your laboratory protocols in protocols.io to enhance the reproducibility of your results. Protocols.io assigns your protocol its own identifier (DOI) so that it can be cited independently in the future. For instructions see: https://journals.plos.org/plosone/s/submission-guidelines#loc-laboratory-protocols. Additionally, PLOS ONE offers an option for publishing peer-reviewed Lab Protocol articles, which describe protocols hosted on protocols.io. Read more information on sharing protocols at https://plos.org/protocols?utm_medium=editorial-email&utm_source=authorletters&utm_campaign=protocols.

We look forward to receiving your revised manuscript.

Kind regards,

José Antonio Ortega, Ph.D.

Academic Editor

PLOS One

Journal Requirements:

When submitting your revision, we need you to address these additional requirements.

1. Please ensure that your manuscript meets PLOS ONE's style requirements, including those for file naming. The PLOS ONE style templates can be found at

https://journals.plos.org/plosone/s/file?id=wjVg/PLOSOne_formatting_sample_main_body.pdf and https://journals.plos.org/plosone/s/file?id=ba62/PLOSOne_formatting_sample_title_authors_affiliations.pdf

2. Please include your full ethics statement in the ‘Methods’ section of your manuscript file. In your statement, please include the full name of the IRB or ethics committee who approved or waived your study, as well as whether or not you obtained informed written or verbal consent. If consent was waived for your study, please include this information in your statement as well.

3. Please upload a copy of Figure 1, to which you refer in your text on page 5, 6, 11, 12. If the figure is no longer to be included as part of the submission please remove all reference to it within the text.

4. If the reviewer comments include a recommendation to cite specific previously published works, please review and evaluate these publications to determine whether they are relevant and should be cited. There is no requirement to cite these works unless the editor has indicated otherwise.

[Note: HTML markup is below. Please do not edit.]

Reviewers' comments:

Reviewer's Responses to Questions

**Comments to the Author**

1. Is the manuscript technically sound, and do the data support the conclusions?

The manuscript must describe a technically sound piece of scientific research with data that supports the conclusions. Experiments must have been conducted rigorously, with appropriate controls, replication, and sample sizes. The conclusions must be drawn appropriately based on the data presented.

Reviewer #1: Yes

Reviewer #2: Yes

**********

2. Has the statistical analysis been performed appropriately and rigorously?

Reviewer #1: Yes

Reviewer #2: Yes

**********

3. Have the authors made all data underlying the findings in their manuscript fully available?

The PLOS Data policy requires authors to make all data underlying the findings described in their manuscript fully available without restriction, with rare exception (please refer to the Data Availability Statement in the manuscript PDF file). The data should be provided as part of the manuscript or its supporting information, or deposited to a public repository. For example, in addition to summary statistics, the data points behind means, medians and variance measures should be available. If there are restrictions on publicly sharing data—e.g. participant privacy or use of data from a third party—those must be specified.

Reviewer #1: No

Reviewer #2: Yes

**********

4. Is the manuscript presented in an intelligible fashion and written in standard English?

PLOS ONE does not copyedit accepted manuscripts, so the language in submitted articles must be clear, correct, and unambiguous. Any typographical or grammatical errors should be corrected at revision, so please note any specific errors here.

Reviewer #1: No

Reviewer #2: Yes

**********

5. Review Comments to the Author

Please use the space provided to explain your answers to the questions above. You may also include additional comments for the author, including concerns about dual publication, research ethics, or publication ethics. (Please upload your review as an attachment if it exceeds 20,000 characters)

Reviewer #1: Comment to authors

• The title and abstract emphasize "sexually active women," which is a crucial and relevant sample. However, the Introduction and parts of the Discussion frequently cite national mCPR statistics for "all women" or "married women" from the LDHS reports. This creates a slight disconnect. The manuscript would be strengthened by consistently framing the entire narrative around the specific population of interest (sexually active women) and clarifying how/why trends for this group might differ from national averages

• The paper’s aims are broadly stated but could be sharpened. At present, the Introduction mixes global FP trends, Lesotho programme context, and general SSA literature without clearly articulating the specific research questions. In the last paragraph of the Introduction, explicitly list 2–3 concrete objectives

• You classify covariates into “predisposing”, “enabling”, and “need” factors but do not explicitly cite or connect this to a conceptual framework (e.g., Andersen’s behavioral model). This weakens the health services / health economics framing. Explicitly state that you use Andersen’s model (or similar) to structure covariates, and briefly justify the classification (e.g. wealth as enabling, parity as need, etc.), with a reference.

• You repeatedly refer to “sexually active women”, but you do not define how sexual activity is operationalised (e.g. “had sex in the past 4 weeks”, “past 3 months”, or “past 12 months”). This is critical because DHS distinguishes between “ever had sex”, “had sex in last 4 weeks”, etc., and your choice affects both prevalence and comparability over time. In Methods, explicitly state the exact DHS variable(s) used (e.g. `v525` or `v536`) and the time window for “sexually active”, and confirm that the definition is comparable across all four surveys.

• The Introduction says: “contraceptive use among sexually active women… rose from 46.2% in 2004… to 72.1% in 2014… 66.5% in 2023/24 [15]”. But your results (Figure 1, Table 2) show 36.1% in 2004 and 62.7% in 2023. These cannot both be correct for the same population and outcome definition.

• The justification for using the Heckley et al. (2016) method over the more common Wagstaff decomposition is appreciated. However, the explanation is dense and could be more accessible. A clearer, concise summary of the key advantage ("it accounts for the covariance between health and socioeconomic rank") in the main text would be helpful.

• You list methods included in “modern contraception”, which is good. However, DHS definitions evolve over time (e.g. addition of emergency contraception; classification of Lactational Amenorrhea Method). You should state whether your classification is harmonized across survey waves and, if any recoding was done, how. Add a sentence clarifying whether the list of modern methods is identical in all four LDHS rounds and, if not, how discrepancies were addressed.

• In Table 3, the compositional (E) contributions of wealth categories are extremely small (on the order of ±0.0001–0.0014; less than 1% each), yet the Discussion states that “improvements in household wealth contributed significantly” to contraceptive use trends. In contrast, the major compositional contributors are education (especially secondary and higher) and visits to health facilities. Tone down claims about wealth composition driving the trend. Make clear that wealth appears more important in the coefficients component (C)—i.e. changes in the strength of association between wealth and contraceptive use—rather than in shifts in the wealth distribution.

• You list methods included in “modern contraception”, which is good. However, DHS definitions evolve over time (e.g. addition of emergency contraception; classification of Lactational Amenorrhea Method). You should state whether your classification is harmonized across survey waves and, if any recoding was done, how. Add a sentence clarifying whether the list of modern methods is identical in all four LDHS rounds and, if not, how discrepancies were addressed.

• The categorization of "Predisposing, Enabling, Need" factors is standard but not fully utilized in the interpretation. The decomposition results could be discussed through this lens to provide a more structured understanding of what types of factors (e.g., enabling vs. predisposing) drove changes.

• The Blinder-Oaxaca result that 90% of the increase is due to the "coefficients" component (labeled as "contraceptive behaviour change") is a central finding. This term requires more precise definition and interpretation. Does it reflect changes in the *effect* of characteristics (e.g., the impact of being rural changed over time), changes in unobserved factors (e.g., norms, program quality), or both? The discussion of this point (page 16) is good but should be expanded and linked more explicitly to potential drivers like policy changes, service integration (e.g., with HIV programs), and shifts in social norms.

• You report EI values of 0.2360, 0.1606, 0.0473, and –0.0164 for 2004, 2009, 2014, and 2023 respectively. You interpret 2023 as “pro poor” and as inequality having “disappeared”. Yet the magnitude is very small and no standard errors/95% CIs are given for the EI itself. Provide standard errors and 95% CI (or p values) for each EI estimate.

• Figure 3: The caption mentions "three concentration curves" but four are described (2004, 2009, 2014, 2023). Please correct. Ensure the figure is of publication quality with a clear legend.

• If the 2023 EI is not statistically different from zero, it is more precise to describe it as “no significant wealth-related inequality” rather than “pro-poor inequality”.

• The Discussion notes that in 2004 inequality was “significantly influenced” by age, education, and wealth, whereas in 2023 it was “influenced by district, sex of household head and number of living children”. This is consistent with Table 4 but should be interpreted carefully: RIF regression coefficients indicate association with EI, not necessarily causal contribution. Make clearer that these are associations with the index, and avoid strong causal language (“determine”, “cause”).

• There are several internal inconsistencies in descriptive tables that need careful checking.

• The Discussion is rich in cross country comparisons but could be more concise and more directly focused on implications for Lesotho’s policy-making: targeting districts with residual inequality, tailoring outreach to older, high parity women, strengthening community health worker programmes, etc.

• The discussion appropriately cites regional literature but would benefit from deeper integration of Lesotho's unique context. Brief mentions of HIV integration and community health workers are good starts. Elaborate more on specific national policies (e.g., National Strategic Development Plan II, RMNCAH strategy), programs, or health system changes that might explain the dramatic catch-up in rural areas and among poorer women between 2014 and 2023.

• The limitations section is adequate but should be expanded.

Minors:

• The manuscript is generally understandable but contains numerous grammatical errors, typographical errors, and long, sometimes repetitive paragraphs (especially in the Discussion).

• Phrases such as “coul dbe explained by” and “las 12 months” should be corrected.

• Several references are problematic. For example, reference [35] is a paper on dissolved organic matter (DOM) in boreal forest soils, which is clearly unrelated to inequality analysis and looks like a miscitation.

• Boadu (2022) appears more than once in the reference list.

• Some in text references (e.g. “WDW”) are not fully defined (presumably Wagstaff, van Doorslaer, Watanabe).

• Use consistent terms for wealth groups: e.g. “poorest, poorer, middle, richer, richest” throughout, both in text and tables

Reviewer #2: The article is original and meets academic writing requirements with clear statistics and description of findings. Only minor (typos) and deletion under the methodology are brought to the attention of authors.

**********

6. PLOS authors have the option to publish the peer review history of their article (what does this mean?). If published, this will include your full peer review and any attached files.

If you choose “no”, your identity will remain anonymous but your review may still be made public.

**Do you want your identity to be public for this peer review?**  For information about this choice, including consent withdrawal, please see our Privacy Policy.

Reviewer #1: No

Reviewer #2: No

**********

[NOTE: If reviewer comments were submitted as an attachment file, they will be attached to this email and accessible via the submission site. Please log into your account, locate the manuscript record, and check for the action link "View Attachments". If this link does not appear, there are no attachment files.]

To ensure your figures meet our technical requirements, please review our figure guidelines: https://journals.plos.org/plosone/s/figures

You may also use PLOS’s free figure tool, NAAS, to help you prepare publication quality figures: https://journals.plos.org/plosone/s/figures#loc-tools-for-figure-preparation.

NAAS will assess whether your figures meet our technical requirements by comparing each figure against our figure specifications.

Attachment Submitted filename: PLOS ONE REVIEWS.docx

# Author comment: Author response to Decision Letter 1

**DOI:** 10.1371/journal.pone.0352594.r002

24 Apr 2026

Dear Editor,

A detailed Rebuttal letter has been included among the submission documents. The letter provides detailed processes of how the reviewers comments have been addressed.

Attachment Submitted filename: Response to Reviewers.docx

# Peer review: Decision Letter 1

**DOI:** 10.1371/journal.pone.0352594.r003

**Authors:** José Antonio Ortega, José Antonio Ortega

11 Jun 2026

Understanding the Drivers of Modern Contraceptive Use Trend Change and Inequalities Among Sexually Active Women in Lesotho: A Decomposition Analysis (2004–2023)

PONE-D-25-62366R1

Dear Dr. Phiri,

We’re pleased to inform you that your manuscript has been judged scientifically suitable for publication and will be formally accepted for publication once it meets all outstanding technical requirements.

Within one week, you’ll receive an e-mail detailing the required amendments. When these have been addressed, you’ll receive a formal acceptance letter and your manuscript will be scheduled for publication.

An invoice will be generated when your article is formally accepted. Please note, if your institution has a publishing partnership with PLOS and your article meets the relevant criteria, all or part of your publication costs will be covered. Please make sure your user information is up-to-date by logging into Editorial Manager at Editorial Manager® and clicking the ‘Update My Information' link at the top of the page. For questions related to billing, please contact billing support.

If your institution or institutions have a press office, please notify them about your upcoming paper to help maximize its impact. If they’ll be preparing press materials, please inform our press team as soon as possible -- no later than 48 hours after receiving the formal acceptance. Your manuscript will remain under strict press embargo until 2 pm Eastern Time on the date of publication. For more information, please contact onepress@plos.org.

Kind regards,

José Antonio Ortega, Ph.D.

Academic Editor

PLOS One

Additional Editor Comments (optional):

From the two previous reviewers, reviewer 2 proposes to accept. Reviewer 1, who had provided very detailed comments, was not available. However, the academic editor has observed that all the comments have been addressed in the -also detailed- response and the new manuscript, therefore, the manuscript is much enhanced and ready for publication.

Reviewers' comments:

Reviewer's Responses to Questions

**Comments to the Author**

1. If the authors have adequately addressed your comments raised in a previous round of review and you feel that this manuscript is now acceptable for publication, you may indicate that here to bypass the “Comments to the Author” section, enter your conflict of interest statement in the “Confidential to Editor” section, and submit your "Accept" recommendation.

Reviewer #2: All comments have been addressed

**********

2. Is the manuscript technically sound, and do the data support the conclusions?

The manuscript must describe a technically sound piece of scientific research with data that supports the conclusions. Experiments must have been conducted rigorously, with appropriate controls, replication, and sample sizes. The conclusions must be drawn appropriately based on the data presented.

Reviewer #2: Yes

**********

3. Has the statistical analysis been performed appropriately and rigorously?

Reviewer #2: Yes

**********

4. Have the authors made all data underlying the findings in their manuscript fully available?

The PLOS Data policy requires authors to make all data underlying the findings described in their manuscript fully available without restriction, with rare exception (please refer to the Data Availability Statement in the manuscript PDF file). The data should be provided as part of the manuscript or its supporting information, or deposited to a public repository. For example, in addition to summary statistics, the data points behind means, medians and variance measures should be available. If there are restrictions on publicly sharing data—e.g. participant privacy or use of data from a third party—those must be specified.

Reviewer #2: Yes

**********

5. Is the manuscript presented in an intelligible fashion and written in standard English?

PLOS ONE does not copyedit accepted manuscripts, so the language in submitted articles must be clear, correct, and unambiguous. Any typographical or grammatical errors should be corrected at revision, so please note any specific errors here.

Reviewer #2: Yes

**********

6. Review Comments to the Author

Please use the space provided to explain your answers to the questions above. You may also include additional comments for the author, including concerns about dual publication, research ethics, or publication ethics. (Please upload your review as an attachment if it exceeds 20,000 characters)

Reviewer #2: After the initial review, the manuscript looks good for publication. Grammatical and spelling errors and have been corrected with attention. The methodology, findings and flow of the paper are good.

**********

7. PLOS authors have the option to publish the peer review history of their article (what does this mean?). If published, this will include your full peer review and any attached files.

If you choose “no”, your identity will remain anonymous but your review may still be made public.

**Do you want your identity to be public for this peer review?**  For information about this choice, including consent withdrawal, please see our Privacy Policy.

Reviewer #2: No

**********

# Editor report: Acceptance letter

**DOI:** 10.1371/journal.pone.0352594.r004

**Authors:** José Antonio Ortega, José Antonio Ortega

PONE-D-25-62366R1

PLOS One

Dear Dr. Phiri,

I'm pleased to inform you that your manuscript has been deemed suitable for publication in PLOS One. Congratulations! Your manuscript is now being handed over to our production team.

At this stage, our production department will prepare your paper for publication. This includes ensuring the following:

* All references, tables, and figures are properly cited

* All relevant supporting information is included in the manuscript submission,

* There are no issues that prevent the paper from being properly typeset

You will receive further instructions from the production team, including instructions on how to review your proof when it is ready. Please keep in mind that we are working through a large volume of accepted articles, so please give us a few days to review your paper and let you know the next and final steps.

Lastly, if your institution or institutions have a press office, please let them know about your upcoming paper now to help maximize its impact. If they'll be preparing press materials, please inform our press team within the next 48 hours. Your manuscript will remain under strict press embargo until 2 pm Eastern Time on the date of publication. For more information, please contact onepress@plos.org.

You will receive an invoice from PLOS for your publication fee after your manuscript has reached the completed accept phase. If you receive an email requesting payment before acceptance or for any other service, this may be a phishing scheme. Learn how to identify phishing emails and protect your accounts at https://explore.plos.org/phishing.

If we can help with anything else, please email us at customercare@plos.org.

Thank you for submitting your work to PLOS ONE and supporting open access.

Kind regards,

PLOS ONE Editorial Office Staff

on behalf of

Dr. José Antonio Ortega

Academic Editor

PLOS One
